# Trends in COPD severe exacerbations, and all-cause and respiratory mortality, before and after implementation of newer long-acting bronchodilators in a large population-based cohort

**DOI:** 10.1186/s12890-024-03277-2

**Published:** 2024-09-13

**Authors:** Charles-Antoine Guay, François Maltais, Claudia Beaudoin, Pierre-Hugues Carmichael, Elhadji Anassour Laouan Sidi, Laurie Perreault, Caroline Sirois, Steeve Provencher

**Affiliations:** 1 Centre de Recherche de l’Institut universitaire de cardiologie et de pneumologie de Québec, Université Laval, 2725, chemin Sainte-Foy, Québec, QC G1V 4G5 Canada; 2 Institut national de santé publique du Québec, Québec City, Canada; 3 Département des sciences de la santé communautaire, Faculté de médecine et des sciences de la santé, Université de Sherbrooke, Sherbrooke, Canada; 4 Department of Medicine, Université Laval, Québec City, Canada; 5 Centre d’excellence sur le vieillissement de Québec, Québec, Canada; 6 Faculty of pharmacy, Université Laval, Québec City, Canada

**Keywords:** COPD exacerbations, Fixed-dose combination, Interrupted time series

## Abstract

**Background:**

Little is known about the trends in morbidity and mortality at the population level that followed the introduction of newer once-daily long-acting bronchodilators for COPD. The purpose of the study was to evaluate whether the availability of new bronchodilators was associated with changes in the temporal trends in severe COPD exacerbations and mortality between 2007 and 2018 in the older population with COPD; and whether this association was homogeneous across sex and socioeconomic status classes.

**Methods:**

We used an interrupted time-series and three segments multivariate autoregressive models to evaluate the adjusted changes in slopes (i.e., trend effect) in monthly severe exacerbation and mortality rates after 03/2013 and 02/2015 compared to the tiotropium period (04/2007 to 02/2013). Cohorts of individuals > 65 years with COPD were created from the nationally representative database of the Quebec Integrated Chronic Disease Surveillance System in the province of Quebec, Canada. Whether these trends were similar for men and women and across different socioeconomic status classes was also assessed.

**Results:**

There were 130,750 hospitalizations for severe exacerbation and 104,460 deaths, including 24,457 (23.4%) respiratory-related deaths, over the study period (928,934 person-years). Significant changes in trends were seen after 03/2013 for all-cause mortality (-1.14%/month;95%CI -1.90% to -0.38%), which further decreased after 02/2015 (-1.78%/month;95%CI -2.70% to -0.38%). Decreases in respiratory-related mortality (-2.45%/month;95%CI -4.38% to -0.47%) and severe exacerbation (-1,90%/month;95%CI -3.04% to -0.75%) rates were only observed after 02/2015. These observations tended to be more pronounced in women than in men and in higher socioeconomic status groups (less deprived) than in lower socioeconomic status groups (more deprived).

**Conclusions:**

The arrival of newer bronchodilators was chronologically associated with reduced trends in severe exacerbation, all-cause and respiratory-related mortality rates among people with COPD > 65 years. Our findings document population benefits on key patient-relevant outcomes in the years following the introduction of newer once-daily long-acting bronchodilators and their combinations, which were likely multifactorial. Public health efforts should focus on closing the gap between lower and higher socioeconomic status groups.

**Supplementary Information:**

The online version contains supplementary material available at 10.1186/s12890-024-03277-2.

## Background

Chronic obstructive pulmonary disease (COPD) remains one of the leading causes of morbidity and mortality worldwide. With an estimated global prevalence of 251 million cases [1], COPD is expected to become the third leading cause of death by 2030 [2]. In the United States alone, COPD generates more than $40 billion in direct annual health care costs, with near 700,000 hospitalizations for severe acute exacerbations (AE-COPD) responsible for most of the economic burden [3–5].

The therapeutic arsenal for COPD has evolved greatly in the last few decades. Long-acting antimuscarinics (LAMAs) and beta_2_-agonist (LABAs) medications were first introduced in the early 2000’s. More recently, once-daily ultra-long-acting beta_2_-agonists (ultra-LABAs), newer LAMAs, and fixed-dose combinations (FDCs) of ultra-LABA/LAMA have been introduced. Several clinical studies and meta-analyses have proven the efficacy of each molecule for improving lung function or preventing future COPD exacerbations [6–8]. However, the addition of ultra-LABAs and FDCs have not consistently prevented severe AE-COPD compared the previously available LAMA (tiotropium) and none of the trials documented survival benefit [9–11].

Lower socioeconomic status has been associated with increased hospitalization rates and mortality in patients with COPD [12, 13]. The influence of socioeconomic deprivation on adherence to prescribed medications in COPD has also been questioned [13]. There is also a growing body of evidence supporting important sex and gender differences in COPD susceptibility, disease manifestations, histological pattern and access to new bronchodilators [14–17]. In the context of scarce resources and increasing health inequalities in developed countries, assessment of population effectiveness of new ultra-LABAs/newer-LAMAs and FDCs is needed to justify an extended use of these expansive new molecules and to inform clinical decision-making. Understanding the influence of socioeconomic status and sex/gender is also critical to ensure a fair distribution of public health resources.

We aimed to assess whether the introduction of new bronchodilators influenced the temporal trends in severe COPD exacerbations and mortality among the older COPD population from 2007 to 2018, and to determine if this association varied across different sexes and socioeconomic status classes using population-based data.

## Methods

### Data source and study design

We used interrupted time-series to compare monthly severe AE-COPD, all-cause and respiratory mortality rates following the introduction of newer once-daily long-acting bronchodilators among older adults with COPD. Data were obtained from the Quebec Integrated Chronic Disease Surveillance System (QICDSS) [18]. Since 1996, this surveillance system has enabled chronic disease monitoring in Quebec, including COPD, by linking provincial health services administrative data using a unique patient identifier. The QICDSS comprises five health administrative databases that include information on registration plans, death registries, and physician service fees. It also contains pharmaceutical claims records obtained from the Provincial health insurance board (Quebec Health Insurance Board [RAMQ]) and it includes hospital discharge abstract records from the hospitalisation databases (Maintenance and Use of Data for the Study of Hospital Clientele [MED-ECHO]) owned by the Quebec Ministry of Health and housed at RAMQ. Demographic data includes place of residence, age, sex and a neighborhood-level social and material deprivation index as an ecological substitute of the socioeconomic status [19]. Data in the QICDSS are updated every fiscal year (fiscal year begins April 1rst and ends March 31rst). Hospital discharge records include the admission diagnosis, primary diagnosis and up to 29 secondary diagnoses coded using the Canadian adaptation of the Tenth Revision of the International Classification of Diseases (ICD-10-CA) system. As the province of Quebec has a universal healthcare system, the QICDSS includes medical records for over 99% of the population ensuring extensive data availability for surveillance and research purposes. The pharmaceutical database provides information on drugs dispensed to individual covered by the province’s public drug insurance plan. In Quebec, drug insurance is mandatory, and all individuals aged 65 and older are eligible for the public drug plan. However, about 10% of these individuals opt for private insurance or receive medication through their nursing home instead.

Finally, the QICDSS incorporates the material and social deprivation index, a composite index that measures material and social deprivation at the ecological level, serving as a proxy for socioeconomic status [19]. It integrates indicators from the Canadian census such as income, employment, education, and family structure to provide a comprehensive assessment of deprivation within communities. Each dimension of the index is divided in quintile depending on the score for each component. A higher deprivation index is associated with a lower SES which has been previously validated in the adult population in Quebec [19].

This study was reviewed and approved by the Laval University institutional review board (2021 − 271).

### Study population and periods

We restricted our population to individuals 66 years and older to ensure comprehensive coverage from our dataset (> 90% of the older population in Quebec is covered by the public health and drug plans) [18]. It also maximised the positive predictive value of COPD case-definition since the prevalence of this disease increases with age [20]. A diagnosis of COPD was defined as either ≥ 1 diagnosis of COPD registered within the hospitalization database (primary or secondary, Supplementary Table 1); or ≥ 3 diagnoses of COPD registered in the physician claims database within the last three years. This validated algorithm has a sensitivity of 59.3% (95%CI:49.7–68.4), a specificity of 95.4% (95%CI:92.6–97.4), and a positive predictive value of 81.7% (95%CI:71.6–89.4) in adults over the age of 35 [21]. No exclusion criteria were applied. We constructed monthly cohorts using data at the individual level (Supplementary Fig. 1) based on the individual fulfilment of the case definition for that month, allowing us to further reduce the number of false positive cases.

We used data from April 1st 2007 to August 31st 2018 to avoid historical bias from the transition in the ICD revision used in the QICDSS (from ICD-9 to ICD-10 codes in 2006), from the introduction of the ultra-LABA/LAMA/corticosteroids single inhaler in Quebec (09/2018) and from the COVID-19 pandemic [18, 22]. Given that ultra-LABAs and newer-LAMAs were launched simultaneously, three distinct segments were determined by the date when ultra-LABAs and FDCs were first approved and covered by the public drug plan, separating the tiotropium period (04/2007 to 02/2013), the post-ultra-LABAs (03/2013 to 01/2015) and the post-FDCs (02/2015 to 08/2018) periods.

### Outcomes

The primary outcome was the monthly rate of severe AE-COPD (requiring hospitalization). Only cases with a primary diagnosis of AE-COPD in the hospitalization database were retained in primary analyses (Supplementary Table 1). A washout period of 14 days between two severe AE-COPD was used to avoid misclassification of relapsing AE-COPD. Secondary outcomes included monthly rates of all-cause and respiratory mortality, the later defined as a primary cause of death associated with diseases of the respiratory system in the death registry.

### Statistical analysis

For each outcome, we calculated monthly incidence rates by dividing the number of new severe AE-COPD, all-cause or respiratory-related deaths by the person-time at risk over the month. We then used three segments multivariate autoregressive models to evaluate changes in slopes (i.e., trend effect) after 03/2013 and 04/2015 compared to the tiotropium period (04/2007 to 02/2013). We did not test for an abrupt change in the level of severe AE-COPD after ultra-LABAs/newer-LAMAs and FDCs introduction since they were introduced progressively after their arrival on the market [23]. We included a season term in the model to account for a simple seasonality pattern of peak in AE-COPD incidence during winter and nadir in summer [14, 24]. Major confounding factors identified graphically using a directed acyclic graph of the relationship between the introduction of ultra-LABAs/newer LAMAs and FDCs and severe COPD exacerbations were accounted for in the regression analyses. They included age, sex, past exacerbations, comorbidities prevalence, medications for COPD (including inhaled corticosteroids in monotherapy/combination), material deprivation, social deprivation and region of residence. Other individual-level confounders identified graphically were not included in the statistical analyses because of their unavailability in the data sources (e.g., smoking status, COPD severity, occupational exposure and vaccination status) and their tendency to change only slowly over time (Supplementary Fig. 2). In the statistical models, age is defined as the average age of individuals in the monthly cohorts, based on their age at the start of the fiscal year. Sex is represented by the proportion of women in these cohorts. Past exacerbations are quantified by the proportion of individuals who have had at least one moderate to severe exacerbation in the past year, with moderate exacerbations defined as those requiring antibiotic or prednisone treatment for 5 to 14 consecutive days in the physician claims database. Comorbidities prevalence is the average number of comorbidities among cohort members (the list of diseases of interest and ICD-codes are available in Supplementary Table 1). COPD medication use includes short-acting and long-acting bronchodilators, inhaled corticosteroids (either as monotherapy or in combination), smoking cessation medication, and macrolide prophylaxis. Drug utilization was defined by at least one billing in the previous 12 months in the pharmaceutical database. Material and social deprivation are measured by the proportion of individuals in each quintile of their respective indices. Finally, region of residence is defined as the proportion of the population living in rural areas. Terms with marginal month-to-month variation were excluded from the final models to avoid collinearity issues. We conducted stratified analyses according to sex and socioeconomic status quintiles using the material deprivation index incorporated in the QICDSS [18, 19].

In sensitivity analyses, we used a second case definition maximising sensitivity over specificity, defining COPD as either ≥ 1 diagnosis of COPD registered to the hospitalization database or ≥ 1 diagnosis of COPD registered to the physician claims database in an unspecified time period. This algorithm has been validated with a sensitivity of 85.0% (95%CI:77.0–91.0), a specificity of 78.4% (95%CI:73.6–82.7) and a positive predictive value of 57.5% (95%CI:49.6–65.1) in adults over the age of 35^21^. We also performed interrupted time-series analyses with different change points to test the robustness of the model. Based on the estimated number needed to treat to prevent one severe AE-COPD of 11 over a 12-month period [8], the selected change points correspond to the date when 10% of COPD cases were ultra-LABAs/newer-LAMAs users (04/2015) or FDCs users (02/2018). All analyses were performed with SAS statistical software (version 9.4; SAS institute, Inc), with 95% confidence-intervals computed using a 5% level of statistical significance. Complete data analyses were performed using population aggregates at each time point, with the QICDSS providing extensive coverage of our studied population and only minimal (< 10%) missing individual data [19]. We did not adjust for multiplicity.

This study followed the RECORD-PE reporting guidelines for pharmacological research using observational routinely collected health data [25].

## Results

### Study population

**Fig. 1:**
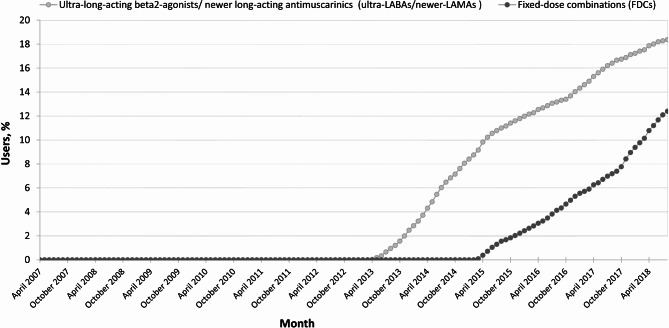
Proportion of ultra-LABAs/newer-LAMAs and FDC users between April 2007 and August 2018. The first ultra-LABAs (indacaterol) was introduced on the list of drugs covered by the public drug plan in February 2013 and was first reimbursed in March 2013. The first FDC (indacaterol/glycopyrronium) was introduced on the list of drugs covered by the public drug plan in February 2015 and was first reimbursed in February 2015. Listed ultra-LABAs/newer-LAMAs in the public drug plan: indacaterol (ultra-LABA), glycopyrronium and umeclidinium (newer-LAMAs). Listed FDCs in the public drug plan: indacaterol/glycopyrronium, vilanterol/umeclidinium, olodaterol/tiotropium

There were 130,750 hospitalizations for severe AE-COPDs and 104,460 deaths, including 24,457 (23.4%) respiratory-related deaths, over the study period (928,934 person-years). Characteristics of the study cohorts remained similar over the years, except for slight increases in age, women proportion and mean number of comorbidities (Table 1). The proportion of ultra-LABAs/newer-LAMAs’ and FDCs users increased steadily after 03/2013 and 02/2015, respectively, to reach 18.4% and 13.5% by the end of the study period (Fig. 1). The proportion of ultra-LABAS/newer-LAMAs users tended to be lower in less deprived subgroups (Supplementary Fig. 3), whereas the proportions of ultra-LABAS/newer-LAMAs and FDCs users were numerically lower in women (Supplementary Fig. 4).

**Table 1:** Baseline characteristics of COPD patients between April 2007 and August 2018

	Pre-ultra-LABAs/newer LAMAs period (April 2007- February 2013)	Post-ultra-LABAs/newer LAMAs period (March 2013- January 2015)	Post-FDCs period (February 2015- August 2018)
	*N* = 64,161	*N* = 76,759	*N* = 79,401
Age^a^ (mean ± SD), years	77.6 (± 7.0)	77.9 (± 7.4)	78.0 (± 7.5)
Sex			
Men	33,398 (52.1)	37,900 (49.4)	39,070 (49.2)
Women	30,763 (47.9)	38,859 (50.6)	40,331 (50.8)
Past exacerbation (moderate-to-severe)^b^ in the previous year			
Yes	10,963 (17.1)	11,784 (15.4)	12,517 (15.8)
No	53,198 (82.9)	64,975 (84.6)	66,884 (84.2)
Comorbidities^c^ (mean ± SD)	5.43 (± 3.3)	5.61 (± 3.4)	5.79 (± 3.5)
Diabetes	17,879 (27.9)	23,413 (30.5)	24,581 (31.0)
Heart failure	15,958 (24.9)	17,217 (22.4)	18,189 (22.9)
Hypertension	44,135 (68.8)	54,723 (71.3)	56,933 (71.7)
Obesity	7,007 (10.9)	9,087 (11.8)	10,014 (12.6)
Medication			
Short-acting bronchodilators	30,610 (47.7)	47,035 (61.3)	48,140 (60.6)
SABAs	28,502 (44.4)	46,162 (60.1)	47,377 (59.7)
SAMAs	7,772 (12.1)	4,954 (6.5)	4,200 (5.3)
Long-acting bronchodilators	23,220 (36.2)	40,400 (52.6)	40,389 (50.8)
LABAs^d^	14,105 (22.0)	6,666 (8.7)	5,768 (7.3)
Tiotropium	14,720 (22.9)	38,448 (50.1)	38,639 (48.7)
Ultra-LABAs/Newer-LAMAs	0 (0.00)	0 (0.00)	6,949 (8.75)
Ultra-LABAs	0 (0.00)	0 (0.00)	2,993 (3.77)
Newer-LAMAs	0 (0.00)	0 (0.00)	5,632 (7.09)
FDCs	0 (0.00)	0 (0.00)	0 (0.00)
ICS	41,326 (64.4)	50,750 (66.1)	51,857 (65.3)
Methylxanthines	3,895 (6.1)	2,236 (2.9)	1,799 (2.3)
Smoking cessation treatments	4,012 (6.3)	6,210 (8.1)	6,465 (8.1)
Material deprivation			
1st quintile (least deprived)	7,379 (11.5)	8,340 (10.9)	7,959 (10.0)
2nd quintile	9,848 (15.3)	11,470 (14.9)	10,492 (13.2)
3rd quintile	11,897 (18.5)	13,720 (17.9)	13,673 (17.2)
4th quintile	14,164 (22.1)	16,089 (21.0)	16,110 (20.3)
5th quintile (most deprived)	16,623 (25.9)	17,946 (23.4)	19,335 (24.4)
Missing values^e^	4,250 (6.6)	9,194 (12.0)	11,832 (14.9)
Region of residence			
Rural (< 10 000 inhabitants)	16,609 (25.9)	19,227 (25.1)	19,560 (24.7)
Urban (≥ 10 000 inhabitants)	47,421 (74.1)	57,428 (74.9)	59,739 (75.3)

aAge at the beginning of the current fiscal year. bModerate exacerbations were defined as COPD exacerbation in the physician claims database requiring antibiotic or prednisone (any dose) treatment for at least five consecutive days and no more than 14 consecutive days. cAverage number of comorbidities in individuals contributing to monthly cohorts. dSalmeterol and formoterol. eSources of missing data include invalid zip codes, cases living in long term care facilities or in nursing homes and areas not cover by the material and social deprivation index (mostly isolated rural regions). Abbreviations: FDC = Fixed dual dose combinations; ICS = Inhaled corticosteroids; LABA = Long-acting beta_2_-agonist; LAMA = Long-acting antimuscarinics; SABA = Short-acting B_2_-agonist; SAMA = Short-acting antimuscarinics; SD = standard deviation

### Severe AE-COPD

**Fig. 2:**
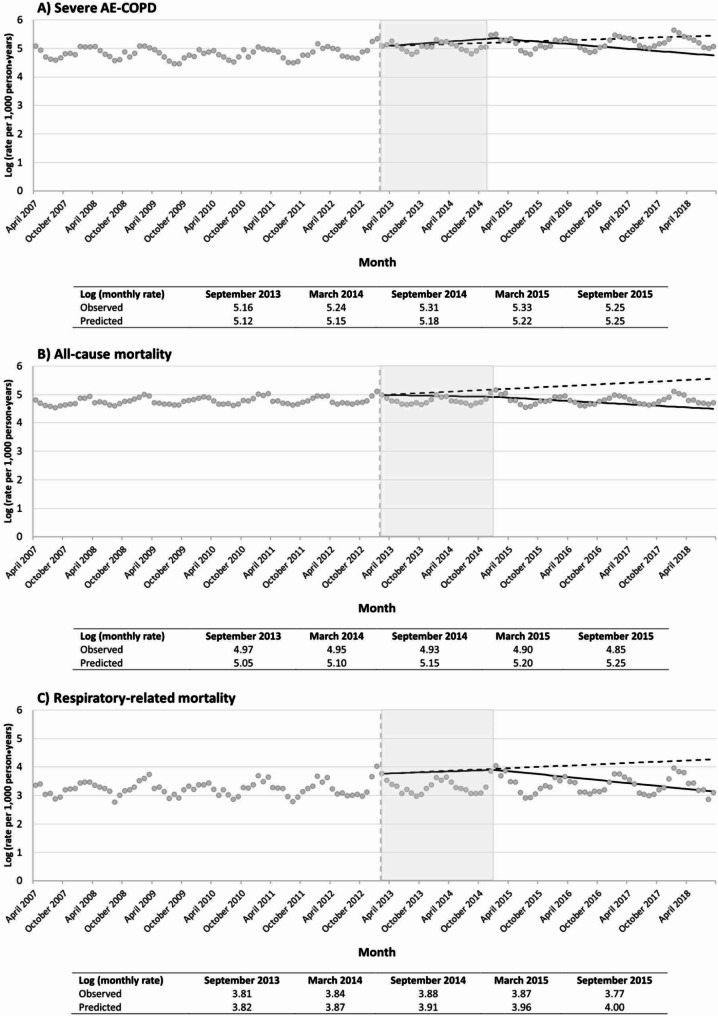
Severe AE-COPD and mortality trends before and after the introduction of ultra-LABAs/newer-LAMAs and FDCs. Vertical dashed line indicates March 2013 when ultra-LABAs/newer LAMAs were introduced in Quebec, Canada. Grey areas show the period from March 2013 (ultra-LABAs/newer-LAMAs’ introduction) to February 2015 (FDCs’ introduction). Solid regression lines show the observed adjusted trends after the introduction of ultra-LABAs/newer-LAMAs and FDCs, whereas dashed regression lines are the predicted adjusted trends after March 2013 assuming no change in the trends observed before introduction of new bronchodilators. AE-COPD = acute exacerbation of chronic obstructive pulmonary disease; ultra-LABA = ultra-long-acting beta2-agonist; LAMA = long-acting antimuscarinic; FDC = fixed-dose combinations

Visual inspection revealed a clear seasonal pattern in monthly severe AE-COPD rate (Fig. 2). An upward trend (+ 0.55%/month;95%CI 0.17–0.94) was observed in severe AE-COPD incidence from 04/2007 to 02/2013 (Table 2). In the segmented regression model, only the introduction of FDCs was associated with significantly sustained decrease in severe AE-COPD rate (-1,90%/month;95%CI -3.04% to -0.75%) (Table 2). Baseline severe AE-COPD rates were higher amongst patients with the lowest socioeconomic status (Table 2, Supplementary Fig. 5), but similar for men and women (Table 3, Supplementary Fig. 6). The reduction tended to be higher in the second and third quintiles of the materially deprived population (Table 2, Supplementary Fig. 5) and in women (Table 3, Supplementary Fig. 6).

**Table 2:** Association between the introduction of ultra-LABAs/newer-LAMAs and FDCs and severe AE-COPD and mortality trends – interrupted time series analyses

			Introduction of ultra-LABAs/newer-LAMAs				Introduction of FDCs						
Outcome	Mean monthly rate per 1,000 person-years in 2007 (95% CI)	Trend before March 2013, % (95% CI)^a^	Monthly % change after March 2013 (95% CI)^a^		P value^a^		Monthly % change after February 2015 (95% CI)^a^			P value^a^			
Overall													
Severe AE-COPD	120.49 (104.95 to 136.02)	0.55 (0.17 to 0.94)	0.66 (-0.14 to 1.45)		0.11		-1.90 (-3.04 to -0.75)			0.001			
All-cause mortality	105.08 (98.69 to 111.47)	0.85 (0.50 to 1.22)	-1.14 (-1.90 to -0.38)		0.004		-1.78 (-2.70 to -0.38)			< 0.001			
Respiratory-related mortality	23.87 (20.57 to 27.16)	0.76 (0.15 to 1.37)	-0.17 (-1.52 to 1.19)		0.80		-2.45 (-4.38 to -0.47)			0.02			
Material deprivation													
1st quintile (least deprived)													
Severe AE-COPD	96.82 (79.96 to 113.68)	0.27 (-0.20 to 0.74)	0.99 (0.04 to 1.93)		0.04		-0.76 (-2.02 to 0.52)			0.24			
All-cause mortality	103.86 (89.00 to 118.73)	0.62 (0.30 to 0.95)	-0.55 (-0.93 to -0.17)		0.005		-0.68 (-1.49 to 0.12)			0.10			
Respiratory-related mortality	23.75 (18.19 to 29.31)	0.63 (-0.13 to 1.40)	-0.43 (-1.42 to 0.57)		0.40		-1.40 (-3.25 to 0.49)			0.14			
2nd quintile													
Severe AE-COPD	112.39 (92.43 to 132.36)	1.14 (0.63 to 1.66)	-1.74 (-2.98 to -0.48)		0.007		-1.01 (-1.92 to -0.09)			0.03			
All-cause mortality	107.02 (96.41 to 117.63)	0.58 (0.14 to 1.02)	-0.90 (-1.98 to 0.20)		0.10		-0.78 (-1.56 to 0.01)			0.05			
Respiratory-related mortality	24.17 (19.90 to 28.45)	1.40 (0.52 to 2.29)	-2.41 (-4.51 to -0.26)		0.03		-1.87 (-3.41 to -0.32)			0.02			
3rd quintile													
Severe AE-COPD	120.15 (104.05 to 136.25)	0.21 (-0.19 to 0.61)	0.64 (-0.41 to 1.70)		0.23		-0.94 (-1.90 to 0.02)			0.06			
All-cause mortality	102.87 (90.32 to 115.41)	0.44 (0.15 to 0.74)	-0.99 (-1.69 to -0.29)		0.006		-0.72 (-1.39 to -0.03)			0.04			
Respiratory-related mortality	22.40 (17.29 to 27.50)	0.31 (-0.19 to 0.82)	-0.18 (-1.46 to 1.12)		0.78		-1.71 (-2.89 to -0.50)			0.01			
4th quintile													
Severe AE-COPD	122.04 (103.39 to 140.68)	0.28 (-0.26 to 0.81)	-0.07 (-1.18 to 1.05)		0.90		-0.41 (-1.47 to 0.66)			0.45			
All-cause mortality	98.64 (91.63 to 105.64)	0.03 (-0.24 to 0.30)	-0.17 (-0.74 to 0.40)		0.56		-0.21 (-0.74 to 0.33)			0.44			
Respiratory-related mortality	21.87 (17.51 to 26.24)	0.09 (-0.45 to 0.63)	-0.19 (-1.28 to 0.91)		0.74		-0.73 (-1.75 to 0.30)			0.16			
5th quintile (most deprived)													
Severe AE-COPD	134.75 (120.74 to 148.75)	-0.31 (-0.59 to -0.03)	1.29 (0.68 to 1.90)		< 0.001		-0.08 (-0.79 to 0.64)			0.83			
All-cause mortality	104.64 (97.01 to 112.27)	0.05 (-0.17 to 0.26)	-0.15 (-0.63 to 0.33)		0.53		-0.21 (-0.74 to 0.33)			0.45			
Respiratory-related mortality	23.74 (19.77 to 27.71)	0.14 (-0.35 to 0.63)	-0.20 (-1.29 to 0.90)		0.72		-0.42 (-1.64 to 0.82)			0.51			

aFinal model adjusted for age, sex, prior exacerbations (moderate-to-severe) in the previous year, comorbidities, current use of short-acting bronchodilators, long-acting bronchodilators, inhaled corticosteroids, smoking cessation medication and macrolide prophylaxis, material deprivation, social deprivation, rural area (< 10 000 inhabitants), seasonality and residual autocorrelation. Individuals with missing socioeconomic status value were excluded from stratified analyses

Abbreviations: AE-COPD = acute exacerbation of chronic obstructive pulmonary disease

**Table 3:** Association between the introduction of ultra-LABAs/newer-LAMAs and FDCs and severe AE-COPD and mortality trends according to sex

					Introduction of ultra-LABAs/newer-LAMAs		Introduction of FDCs		
Outcome	Mean monthly rate per 1,000 person-years in 2007 (95% CI)		Trend before March 2013, % (95% CI)^a^		Monthly % change after March 2013 (95% CI)^a^	P value^a^	Monthly % change after February 2015 (95% CI)^a^	P value^a^	
Overall									
Severe AE-COPD		120.49 (104.95 to 136.02)		0.55 (0.17 to 0.94)	0.66 (-0.14 to 1.45)	0.11	-1.90 (-3.04 to -0.75)	0.001	
All-cause mortality		105.08 (98.69 to 111.47)		0.85 (0.50 to 1.22)	-1.14 (-1.90 to -0.38)	0.004	-1.78 (-2.70 to -0.38)	< 0.001	
Respiratory-related mortality		23.87 (20.57 to 27.16)		0.76 (0.15 to 1.37)	-0.17 (-1.52 to 1.19)	0.80	-2.45 (-4.38 to -0.47)	0.02	
Men									
Severe AE-COPD		121.52 (105.87 to 137.20)		0.59 (0.22 to 0.96)	0.47 (-0,28 to 1.22)	0.22	-1.32 (-2.55 to -0.07)	0.04	
All-cause mortality		115.62 (108.92 to 122.30)		0.64 (0.25 to 1.02)	-1.06 (-1.85 to -0.28)	0.01	-1.67 (-2.82 to -0.52)	0.01	
Respiratory-related mortality		25.96 (22.54 to 29.39)		0.55 (-0.05 to 1.15)	-0.67 (-1.93 to 0.60)	0.29	0.49 (-1.64 to 2.68)	0.65	
Women									
Severe AE-COPD		119.36 (102.86 to 135.85)		0.46 (-0.05 to 0.97)	0.80 (-0.24 to 1.85)	0.13	-2.19 (-3.52 to -0.85)	0.002	
All-cause mortality		93.76 (84.84 to 102.67)		0.76 (0.38 to 1.13)	-0.24 (-0.99 to 0.51)	0.52	-2.31 (-3.38 to -1.24)	< 0.001	
Respiratory-related mortality		21.61 (17.89 to 25.34)		1.27 (0.45 to 2.10)	0.23 (-1.45 to 1.93)	0.79	-5.06 (-7.23 to -2.83)	< 0.001	

aFinal model adjusted for age, sex, prior exacerbations (moderate-to-severe) in the previous year, comorbidities, current use of short-acting bronchodilators, long-acting bronchodilators, inhaled corticosteroids, smoking cessation medication and macrolide prophylaxis, material deprivation, social deprivation, rural area (< 10 000 inhabitants), seasonality and residual autocorrelation

Abbreviations: AE-COPD = acute exacerbation of chronic obstructive pulmonary disease

### Mortality

All-cause and respiratory-related mortality rates were also characterized by seasonality (Fig. 2). Upward trends were observed in both all-cause and respiratory-related mortality rates from 04/2007 to 02/2013. Significant changes in trends were seen after the introduction of ultra-LABAs/newer-LAMAs for all-cause mortality, which further decreased after FDCs’ introduction. A significant reduction in respiratory-related monthly mortality rate was only seen after the introduction of FDCs. All-cause and respiratory-related mortality rates were comparable across the diverse socioeconomic status (Table 2, Supplementary Fig. 5), but higher in men (Table 3, Supplementary Fig. 6). Changes in trends for all-cause and respiratory-related mortality rates tended to differ according to socioeconomic status, with more prominent reductions in all-cause and respiratory mortality seen in higher socioeconomic status (less deprived) groups (Table 2, Supplementary Fig. 5) and in women (Table 3, Supplementary Fig. 6).

### Sensitivity analyses

Similar results were observed when using the case-definition maximising sensitivity over specificity, with reduction in trends for AE-COPD, all-cause and respiratory-related mortality being observed after FDCs introduction only (Supplementary Table 2). Finally, the interrupted time series analysis with change points based on the proportion of users showed significant changes in trends for all outcomes after the proportion of users of ultra-LABAs/newer-LAMAs reached 10% (04/2015), with no additional change in trends after the proportion of users of FDCs reached 10% (02/2018, Table 4).

**Table 4:** Association between the introduction of ultra-LABAs/newer LAMAs and FDCs and severe AE-COPD and mortality trends – change points based on the proportion of users reaching 10% for ultra-LABAs/newer-LAMAs and FDCs

			10% users of ultra-LABAs/newer LAMA					10% users of FDCs					
Outcome	Mean monthly rate per 1,000 person-years in 2007 (95% CI)	Trend before April 2015 (95% CI)^a^		Monthly % change after April 2015 (95% CI)^a^			P value^a^	Monthly % change after February 2018 (95% CI)^a^				P value^a^	
Overall													
Severe AE-COPD	120.49 (104.95 to 136.02)	0.64 (0.36 to 0.92)		-1.23 (-2.40 to -0.04)			0.04	-0.81 (-2.60 to 1.00)				0.37	
All-cause mortality	105.08 (98.69 to 111.47)	0.39 (0.19 to 0.59)		-1.78 (-2.71 to -0.85)			< 0.001	1.72 (-0.04 to 3.51)				0.06	
Respiratory-related mortality	23.87 (20.57 to 27.16)	0.63 (0.19 to 1.08)		-2.42 (-4.38 to -0.42)			0.02	1.17 (-2.12 to 4.56)				0.49	

aFinal model adjusted for age, sex, prior exacerbations (moderate-to-severe) in the previous year, comorbidities, current use of short-acting bronchodilators, long-acting bronchodilators, inhaled corticosteroids, smoking cessation medication and macrolide prophylaxis, material deprivation, social deprivation, rural area (< 10 000 inhabitants), seasonality and residual autocorrelation

Abbreviations: AE-COPD = acute exacerbation of chronic obstructive pulmonary disease

## Discussion

This nationally representative data indicates sustained reduced trends in severe AE-COPD, all-cause and respiratory-related mortality adjusted rates after 02/2015 in patients with COPD over 65 years. Interestingly, the timing of the observed reductions corresponds to both the introduction of FDCs (02/2015) and the 10% milestone in ultra-LABAs/newer-LAMAs users (04/2015).

Several clinical trials and meta-analyses have demonstrated greater lung function improvements and symptom reduction with FDCs compared to ultra-LABAs/newer-LAMAs alone irrespective of disease severity [9, 10, 26–28]. Since lung function and dyspnea persistence strongly predict future exacerbations and mortality, it can be argued that the introduction of FDCs could lead to reduced trends in severe AE-COPD and mortality rates [29]. Yet, head-to-head trials assessing comparative efficacy of ultra-LABAs/newer-LAMAs and FDCs found inconclusive results regarding severe AE-COPD and mortality^9–11−15,28^. The SPARK trial was the first trial specifically designed to assess the efficacy of FDC compared to two LAMAs in preventing exacerbations in moderate-to-severe COPD patients for 64 weeks [10]. The combination of indacaterol/glycopyrronium was associated with a 12% risk reduction in moderate-to-severe exacerbations compared to glycopyrronium alone, whereas no significant difference was noted with tiotropium alone. Subsequent meta-analyses of studies comparing FDCs versus LAMAs resulted in similarly inconclusive results [9, 27], whereas FDCs were associated with a 18% risk reduction moderate-to-severe exacerbations compared to LABAs alone in trials that used a twice-daily LABAs as comparator [27]. Finally, trials comparing FDCs to indacaterol, the only ultra-LABA marketed as monotherapy in the province of Quebec, did not assess exacerbation rates as an outcome [30, 31].

Individual clinical trials most commonly have a limited sample size, precluding valid conclusion regarding the added benefit of novel therapies on relatively infrequent clinical outcomes over already effective therapies. To overcome this limitation, large-scale meta-analyses incorporating data from 51 to 99 studies were recently published [27, 28]. The former suggested that LAMA/LABA may be associated with a small increase in the risk of major adverse cardiovascular events [27], whereas the latter suggested no impact of FDCs on mortality rates [28]. While randomized controlled trials and subsequent meta-analyses are essential to document the efficacy and safety of novel therapies, they are characterized by strict eligibility criteria resulting in participants with limited comorbidities and drug-drug interactions, and a very close clinical follow-up of relatively short duration. For example, there were only 110 total deaths amongst the 101,311 patients contributing to the large-scale meta-analysis described above [28], questioning the external validity of their findings. Conversely, the assessment of real-world effectiveness aims to capture the full spectrum of a disease in the context of the routine delivery of care and may significantly differ from clinical trial-based efficacy [32]. For instance, a real world non-interventional study found a 21% risk reduction in severe AE-COPD with FDCs compared to ultra-LABAs/newer-LAMAs monotherapy after adjusting for disease severity and baseline demographic using propensity score matching [33]. Although residual confounding could not be completely excluded, these results suggest that randomized controlled trials may not provide a definitive answer regarding he comparative effectiveness of FDCs versus to ultra-LABAs/newer LAMAs.

In interrupted time series, observations on a population taken at multiple times before and after an intervention are used to assess a change in trend caused by the intervention. The pre-intervention trend is used to estimate the counterfactual scenario in which the intervention had not occurred, offering the advantage over the non-equivalent group control design for controlling for confounding due to between-group differences and underlying trends in the outcome [34]. It also elegantly avoids confounding by indication, which is a significant challenge in measuring COPD treatment effectiveness in real-world situations, since time is the exposure of interest and is not affected by disease severity [34]. In addition, the routinely collected data allow a more detailed assessment of the longitudinal effects of an intervention than typical clinical trials, in addition to stronger external validity [22, 34]. Nevertheless, time series design can be affected by time-varying confounders that may change rapidly over time. Accordingly, important risk factors for severe AE-COPD were included in our final models to strengthen the robustness of our analyses. Mainly, all statistical models were adjusted for ICS use, either as monotherapy or in combination therapy, to account for the fluctuations in ICS prescription patterns over the study period, reflecting the evolving evidence of their benefits on severe COPD exacerbations and mortality [35]. However, in the absence of randomization, the significant changes in trends we observed cannot be specifically inferred to ultra-LABAs/newer-LAMAs or FDCs. Moreover, interrupted time series may be affected by historical bias when another intervention or event occurs at the time of the time-series interruption and may also affect the outcome of interest [22]. Major potential violators of this assumption including the introduction of single inhaler ultra-LABA/LAMA/inhaled corticosteroids triple therapy and the COVID-19 pandemic occurred after the end of the study period and should not affect our results [35]. Also, the overall management of COPD patients has evolved significantly over the past decades, including pulmonary rehabilitation and self-management programs which have been shown to positively impact the rate of severe AE-COPD and have gained in popularity [36, 37], may have contributed to the overall improvement in COPD outcomes observed in our study [38]. Other significant breakthroughs include an increase in outpatient management of exacerbations, improved smoking cessation programs, and enhanced personalized pharmaceutical approaches based on a better understanding of the risk factors for disease progression [4]. The ecological design of the study makes it impossible to distinguish the independent contribution of each of these factors and the introduction of the new bronchodilators on the observed changes in trends for the whole study period. Nevertheless, the consistency of the sensitivity analyses supports the validity of our results. In addition, our findings suggest a greater impact of the availability of new bronchodilators on severe AE-COPD and respiratory-related mortality compared with all-cause mortality, adding to the biological plausibility of the observed associations.

This work also adds to the existing literature about the association between socioeconomic status and outcomes in COPD and other chronic diseases [38–41], including progressively higher rates of severe AE-COPD rates within low socioeconomic status. We observed a similar or slightly higher proportion of users of new bronchodilators in lower socioeconomic status groups, suggesting similar access to medical care, hospitalization and newer therapies in these patients potentially explained by the universal healthcare system in Canada. Nevertheless, patients in lower socioeconomic groups tended to have smaller reductions in severe AE-COPD, all-cause, and respiratory-related mortality after the introduction of FDCs. Whether this is related to a lower adherence to medications or residual confounding could not be assessed. We also found important sex difference in the trend change in COPD outcomes, with benefit being predominantly observed amongst women. Few clinical studies have assessed the differential treatment response to bronchodilators according to sex and gender [42, 43]. A pooled analysis of six trials from the IGNITE program reported larger improvements in health status and overall symptoms in women than men but authors did not report significant differences in lung function improvements [42]. Although clinical evidence of differences in treatment response is lacking, there is a growing body of evidence supporting important sex and gender differences in COPD disease mechanisms and manifestations [14–16]. In addition of divergent smoking habits, women appear to have a greater susceptibility to COPD with lower tobacco exposure and might be less adherent to medication [15, 16]. Moreover, the histological pattern of COPD differs in women with relatively less emphysema and more small airways disease [17]. These differences could influence treatment response and highlight the need to assess sex-related differences in treatment efficacy in future clinical trials.

This study has some limitations. First, the presence of COPD was not clinically confirmed using spirometry introducing a risk of misclassification bias. Spirometry is essential for accurate COPD diagnosis, and its absence can lead to both overdiagnosis and underdiagnosis of the condition [44]. In the context of our study, the accumulation of false positive cases over time for a lifelong diagnosis like COPD could have biased our inferences about the intervention effect on a population with a high prevalence of individuals not at risk of the outcome. Additionally, without post-bronchodilator spirometry, some false positive cases may have actually had asthma instead of COPD, while others may have had Asthma-COPD Overlap Syndrome (ACOS), with evidence suggesting ACOS could have a distinct phenotype and a different treatment response compared to other COPD patients [45]. However, the impact on our results is likely minimal since we used a consistent case definition throughout the study, minimizing the risk of differential misclassification. To further mitigate the impact of false positive cases, we employed an algorithm with high specificity and positive predictive value [17] and included repeated fulfillment criteria to ensure COPD diagnosis stability over time, thereby minimizing the risk of misclassification between asthma and COPD. Conversely, low sensitivity can lead to missed COPD cases, particularly in low socioeconomic status groups for whom limited healthcare access is still a major determinant of health inequalities [46] or patients with less severe COPD at lower risk of repeated medical consultations and hospitalizations. Over time, improved healthcare access and increased awareness of COPD may have led to the identification of more mild COPD cases, potentially biasing results towards an intervention effect. However, sensitivity analyzes using a case definition with higher sensitivity, which reduced the difference in the number of less severe COPD patients, did not yield different results. Second, we could not assess the impact of the introduction of ultra-LABAs/newer LAMAs, and FDCs in specific subgroups of interest, such as individuals with ACOS, nor according to COPD severity due to the absence of spirometry data or validated algorithms to accurately identify these patients; this should be the subject of further study. Third, interpretation of our results as evidence of individual improvement should be done with caution due to the design of our study and the inherent risk of ecological fallacy. Finally, our results may not be generalizable to younger COPD patients, those with private drug insurance and institutionalized individuals, and to less severe outcomes such as mild to moderate exacerbations. Nevertheless, our study provides reassuring results in an older population that is often underrepresented in clinical trials.

## Conclusion

In conclusion, the introduction of newer long-acting bronchodilators was chronologically associated with reduced adjusted trends in severe AE-COPD, all-cause and respiratory-related mortality rates in the older COPD population. Taken together, our findings suggest potential population benefits on key patient-relevant outcomes in the years following the introduction of ultra-LABAs/newer LAMAs and FDCs in COPD patients > 65 years of age. Whether the changes in trends are due to a higher proportion of ultra-LABA/newer LAMA users, the introduction of FDCs, or simply the overall improvement in COPD management remains to be confirmed. The observed sex- and socioeconomic status-related differences in the changes in these trends support the need for prospective clinical trials adequately powered for sex/gender-specific analyses, as well as public health efforts focused on closing the gap between lower and higher socioeconomic groups.

## Electronic supplementary material

Below is the link to the electronic supplementary material.

Supplementary Material 1

## Data Availability

The datasets generated and analysed during the study are not publicly available due to individual privacy stakes. The data are stored on a secure server and regulated by the Quebec Ministry of Health and the Quebec Health Insurance Board. Access to the data is restricted to authorized personnel only to prevent any sensitive data breaches.

## Abbreviations

ACOS: Asthma-COPD Overlap Syndrome
AE-COPD: Acute exacerbation of COPD
CI: Confidence interval
COPD: Chronic obstructive pulmonary disease
FDC: Fixed dual doses combination
ICD-9: International Classification of Diseases, 9th Revision
ICD-10: International Classification of Diseases, 10th Revision
ICD-10-CA: International Classification of Diseases, 10th Revision, Canada
ICS: Inhaled corticosteroids
LABA: Long-acting beta_2_-agonist
LAMA: Long-acting antimuscarinics
MED-ECHO: Maintenance and Use of Data for the Study of Hospital Clientele
QICDSS: Quebec Integrated Chronic Disease Surveillance System
RAMQ: Quebec Health Insurance Board
SABA: Short-acting B_2_-agonist
SAMA: Short-acting antimuscarinics
ultra-LABA: Ultra-long-acting beta_2_-agonist
