# Supplementary material for: Trends in COPD severe exacerbations, and all-cause and respiratory mortality, before and after implementation of newer long-acting bronchodilators in a large population-based cohort
Source: BMC Pulm Med. 2024 Sep 13;24:450. doi: 10.1186/s12890-024-03277-2 (PMC11401429; doi:10.1186/s12890-024-03277-2)
Supplement: Supplementary file 1 — Supplementary Material 1 [file 12890_2024_3277_MOESM1_ESM.docx]

**Trends in COPD severe exacerbations, and all-cause and respiratory mortality, before and after implementation of newer long-acting bronchodilators in a large population-based cohort** Charles-Antoine Guay, François Maltais, Claudia Beaudoin, Pierre-Hugues Carmichael, Elhadji Anassour Laouan Sidi, Laurie Perreault, Caroline Sirois, and Steeve Provencher

**Supplemental Material 1**

**Supplementary Table 1:** ICD-10 diagnostic codes used to identify COPD cases and comorbidities from the co-morbidity index of Charlson and Elixhauser.

**Supplementary Table 2:** Association between the introduction of ultra-LABAs/newer LAMAs and FDCs and severe AE-COPD and mortality trends – Interrupted time series analyses with a secondary case definition maximising sensitivity over specificity.

**Supplementary Figure 1:** Person-time at risk contribution to monthly open cohorts from COPD cases for primary outcome.

**Supplementary Figure 2:** Severe AE-COPD risk factors and causal pathways.

**Supplementary Figure 3:** Proportion of ultra-LABAs/newer LAMAs and FDC users between April 2007 and August 2018 according to socioeconomic status groups.

**Supplementary Figure 4:** Proportion of ultra-LABAs/newer LAMAs and FDC users between April 2007 and August 2018 according to sex.

**Supplementary Figure 5:** Severe AE-COPD and mortality trends by socioeconomic status.

**Supplementary Figure 6:** Severe AE-COPD and mortality trends according to sex.

This supplemental material has been provided by the authors to give readers additional

information about their work.

**Supplementary material**

**Supplementary Table 1: ICD-10 diagnostic codes used to identify COPD cases and comorbidities from the co-morbidity index of Charlson and Elixhauser^2^.**

| **Medical condition** | **ICD-10 code** |
| --- | --- |
| COPD | J41.0 to J44.9 |
| Congestive heart failure | I09.9, I11.0, I13.0, I13.2, I25.5, I42.0, I42.5 to I42.9, I43, I50, P29.0 |
| Cardiac arrhythmias | I44.1 to I44.3, I45.6, I45.9, I47 to I49, R00.0, R00.1, R00.8, T82.1, Z45.0, Z95.0 |
| Valvular disease | A52.0, I05 to I08, I09.1, I09.8, I34 to I39, Q23.0 to Q23.3, Z95.2 to Z95.4 |
| Pulmonary circulation disorders | I26, I27, I28.0, I28.8, I28.9 |
| Peripheral vascular disorders | I70, I71, I73.1, I73.8, I73.9, I77.1, I79.0, I79.2, K55.1, K55.8, K55.9, Z95.8, Z95.9 |
| Hypertension (combined uncomplicated and complicated) | I10, I11 to I13, I15 |
| Paralysis | G04.1, G11.4, G80.1, G80.2, G81, G82, G83.0 to G83.4, G83.9 |
| Other neurological disorders | G10 to G13, G20 to G22, G25.4, G25.5, G31.2, G31.8, G31.9, G32, G35 to G37, G40, G41, G93.1, G93.4, R47.0, R56 |
| Asthma | J45 to J46 |
| Chronic pulmonary disease (excluding COPD and Asthma) | I27.8, I27.9, J47, J60 to J67, J68.4, J70.1, J70.3 |
| Diabetes, uncomplicated | E10.0, E10.1, E10.9, E11.0, E11.1, E11.9, E12.0, E12.1, E12.9, E13.0, E13.1, E13.9, E14.0, E14.1, E14.9 |
| Diabetes, complicated | E10.2 to E10.8, E11.2 to E11.8, E12.2 to E12.8, E13.2 to E13.8, E14.2 to E14.8 |
| Hypothyroidism | E00 to E03, E89.0 |
| Renal failure | I12.0, I13.1, N18, N19, N25.0, Z49.0 to Z49.2, Z94.0, Z99.2 |
| Liver disease | B18, I85, I86.4, I98.2, K70, K71.1, K71.3 to K71.5, K71.7, K72 to K74, K76.0, K76.2 to K76.9, Z94.4 |
| Peptic ulcer disease, excluding bleeding | K25.7, K25.9, K26.7, K26.9, K27.7, K27.9, K28.7, K28.9 |
| AIDS/HIV | B20 to B22, B24 |
| Lymphoma | C81 to C85, C88, C96, C90.0, C90.2 |
| Metastatic cancer | C77 to C80 |
| Solid tumour without metastasis | C00 to C26, C30 to C34, C37 to C41, C43, C45 to C58, C60 to C76, C97 |
| Rheumatoid arthritis/collagen vascular diseases | L94.0, L94.1, L94.3, M05, M06, M08, M12.0, M12.3, M30, M31.0 to M31.3, M32 to M35, M45, M46.1, M46.8, M46.9 |
| Coagulopathy | D65 to D68, D69.1, D69.3 to D69.6 |
| Obesity | E66 |
| Weight loss | E40 to E46, R63.4, R64 |
| Fluid and electrolyte disorders | E22.2, E86, E87 |
| Blood loss anaemia | D50.0 |
| Deficiency anaemia | D50.8, D50.9, D51 to D53 |
| Alcohol abuse | F10, E52, G62.1, I42.6, K29.2, K70.0, K70.3, K70.9, T51, Z50.2, Z71.4, Z72.1 |
| Drug abuse | F11 to F16, F18, F19, Z71.5, Z72.2 |
| Psychoses | F20, F22 to F25, F28, F29, F30.2, F31.2, F31.5 |
| Depression | F20.4, F31.3 to F31.5, F32, F33, F34.1, F41.2, F43.2 |

|  |  | | **Introduction of ultra-LABAs/newer LAMAs** |  | | **Introduction of FDCs** | |  | | |  |
| --- | --- | --- | --- | --- | --- | --- | --- | --- | --- | --- | --- |
| Outcome | Mean monthly rate per 1,000 person-years in 2007 (95% CI) | Trend before March 2013 (95% CI)^a^ | Monthly % change after March 2013 (95% CI)^a^ | P value^a^ | | Monthly % change after February 2015 (95% CI)^a^ | | P value^a^ | | |  |
| Overall |  |  |  |  |  | |  | |  |  | |
| Severe AE-COPD | 87.47 (75.14 to 99.82) | -0.17 (-0.45 to 0.11) | 0.98 (-0.24 to 2.21) | 0.12 | | -1.83 (-3.31 to -0.34) | | 0.02 | | |  |
| All-cause mortality | 80.13 (75.14 to 85.13) | -0.20 (-0.35 to -0.05) | -0.35 (-1.04 to 0.35) | 0.33 | | -1.16 (-2.07 to -0.24) | | 0.01 | | |  |
| Respiratory-related mortality | 17.31 (14.72 to 19.91) | -0.34 (-0.69 to -0.01) | -0.22 (-1.84 to 1.43) | 0.79 | | -1.83 (-3.93 to 0.31) | | 0.07 | | |  |

**Supplementary Table 2: Association between the introduction of ultra-LABAs/newer LAMAs and FDCs and severe AE-COPD and mortality trends – Interrupted time series analyses with a secondary case definition maximising sensitivity over specificity**

^a^Final model adjusted for age, sex, prior exacerbations (moderate-to-severe) in the previous year, comorbidities, current use of short-acting bronchodilators, long-acting bronchodilators, inhaled corticosteroids, smoking cessation medication and macrolide prophylaxis, material deprivation, social deprivation, rural area (< 10 000 inhabitants), seasonality and residual autocorrelation.

Abbreviations: AE-COPD= acute exacerbation of chronic obstructive pulmonary disease.

| Patients | |  | | | | | | | | | | | | | | | | | | | | | | | | | | | | | | | |
| --- | --- | --- | --- | --- | --- | --- | --- | --- | --- | --- | --- | --- | --- | --- | --- | --- | --- | --- | --- | --- | --- | --- | --- | --- | --- | --- | --- | --- | --- | --- | --- | --- | --- |
| 1 | |  |  |  |  |  |  |  |  |  |  |  |  |  |  |  |  |  |  |  |  |  |  |  |  |  |  |  |  |  |  |  | |
| 2 | |  |  |  |  |  |  |  |  |  |  |  |  |  |  |  |  |  |  |  |  |  |  |  |  |  |  |  |  |  |  |  | |
| 3 | |  |  |  |  |  |  |  |  |  |  |  |  |  |  |  |  |  |  |  |  |  |  |  |  |  |  |  |  |  |  |  | |
| 4 | |  |  |  |  |  |  |  |  |  |  |  |  |  |  |  |  |  |  |  |  |  |  |  |  |  |  |  |  |  |  |  | |
| 5 | |  |  |  |  |  |  |  |  |  |  |  |  |  |  |  |  |  |  |  |  |  |  |  |  |  |  |  |  |  |  |  | |
| 6 | |  |  |  |  |  |  |  |  |  |  |  |  |  |  |  |  |  |  |  |  |  |  |  |  |  |  |  |  |  |  |  | |
| 7 | |  |  |  |  |  |  |  |  |  |  |  |  |  |  |  |  |  |  |  |  |  |  |  |  |  |  |  |  |  |  |  | |
| 8 | |  |  |  |  |  |  |  |  |  |  |  |  |  |  |  |  |  |  |  |  |  |  |  |  |  |  |  |  |  |  |  | |
| 9 | |  |  |  |  |  |  |  |  |  |  |  |  |  |  |  |  |  |  |  |  |  |  |  |  |  |  |  |  |  |  |  | |
|  | | 17 | 18 | 19 | 20 | 21 | 22 | 23 | 24 | 25 | 26 | 27 | 28 | 29 | 30 | 31 | 1 | 2 | 3 | 4 | 5 | 6 | 7 | 8 | 9 | 10 | 11 | 12 | 12 | 14 | 15 | 16 | |
|  | January | | | | | | | | | | | | | | | | February | | | | | | | | | | | | | | | |  |

**Legend:**

|  | Not contributing to the analysis |
| --- | --- |
|  | Person-days at risk of severe AE-COPD for February |
|  | Person-days at risk of severe AE-COPD for January |
|  | Outcome occurrence for February |
|  | Outcome occurrence for January |
|  | Concurrent outcome/censure (death, emigration) |

**Supplementary Figure 1: Person-time at risk contribution to monthly open cohorts from COPD cases for primary outcome.**

| **Risk factors** | **Paths** | **References** |
| --- | --- | --- |
| **Individual level** | | |
| Age | 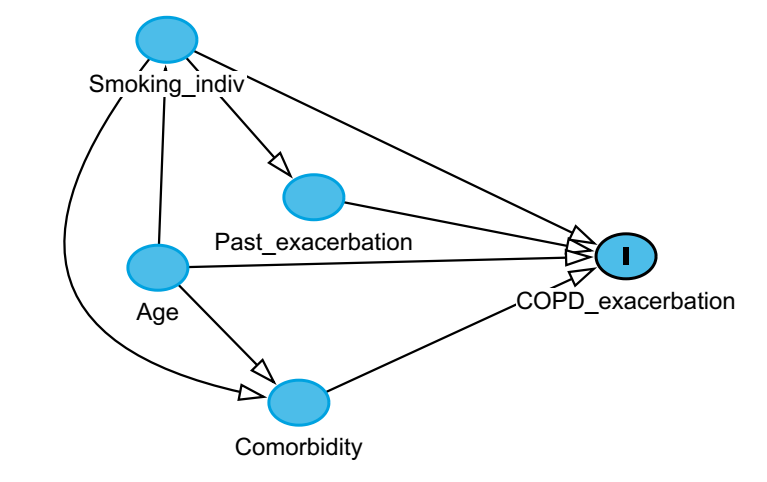 | ^3-5^ |
| Sex | 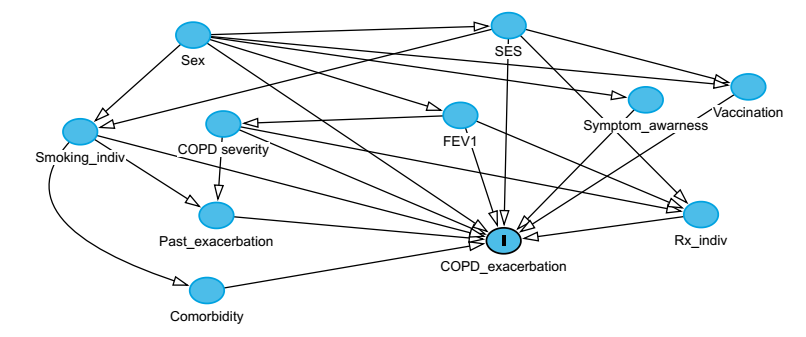 | ^3,4,6^ |
| Smoking status | 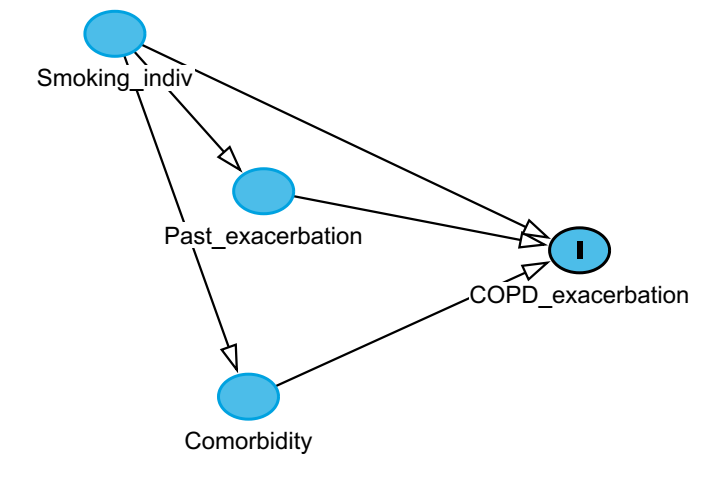 | ^4, 7^ |
| SES | 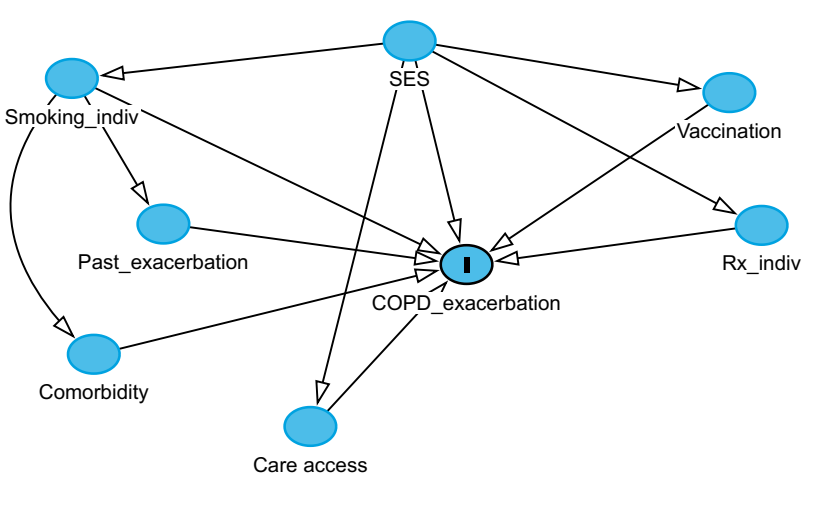 | ^8-12^ |
| COPD severity | 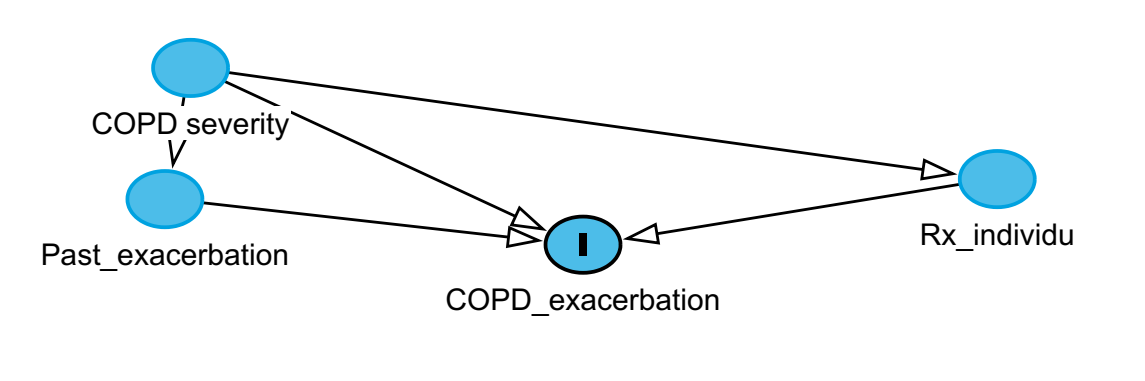 | ^3-6^ |
| Previous exacerbations | 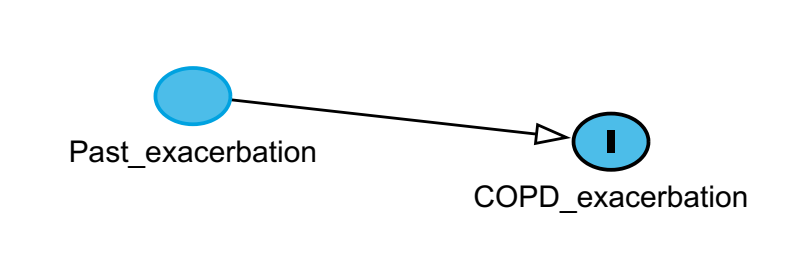 | ^3-6^ |
| FEV_1_ | 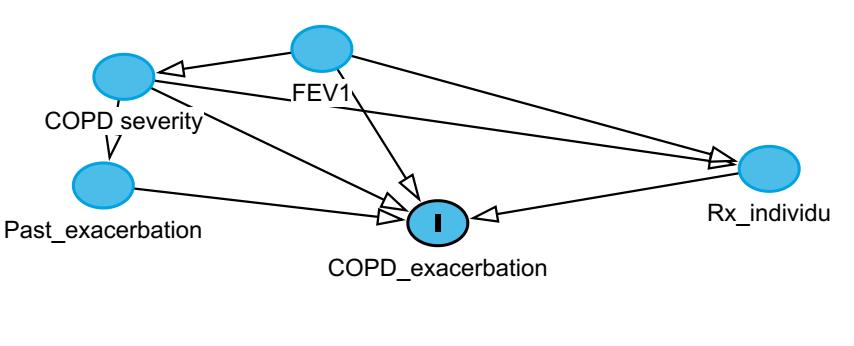 | ^3-6, 14-16^ |
| Pharmacotherapy | 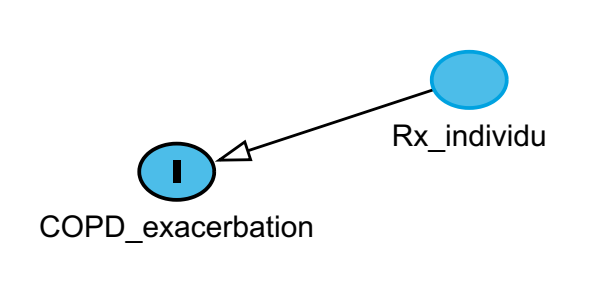 | ^16-21^ |
| Comorbidity | 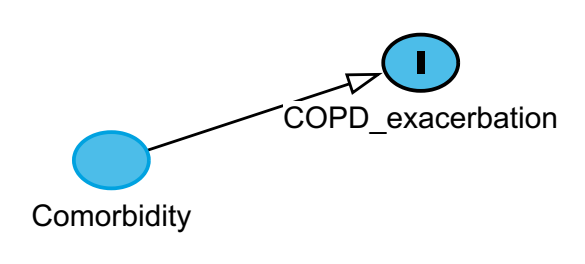 | ^22-25^ |
| Vaccination status | 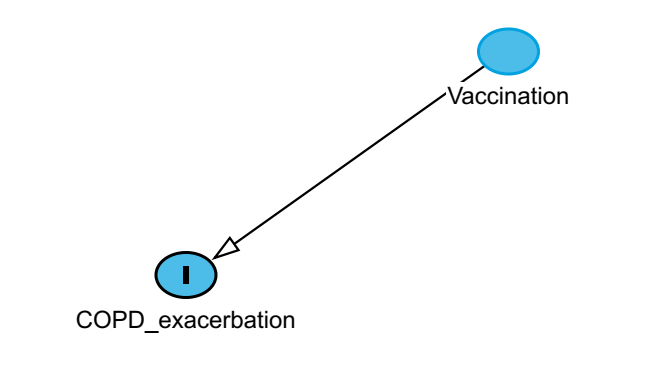 | ^26-28^ |
| Occupational exposure | 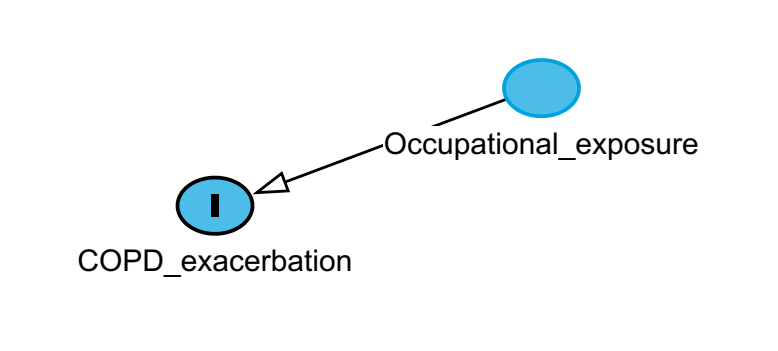 | ^29-30^ |
| **Populational level** | | |
| Seasonality | 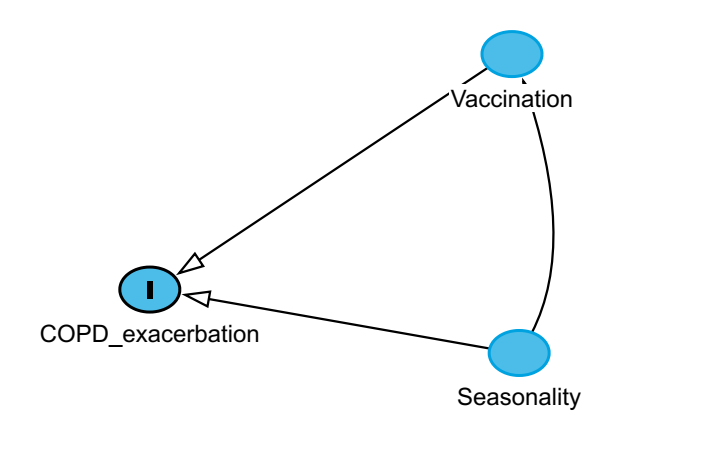 | ^31,32^ |
| Geographical region | 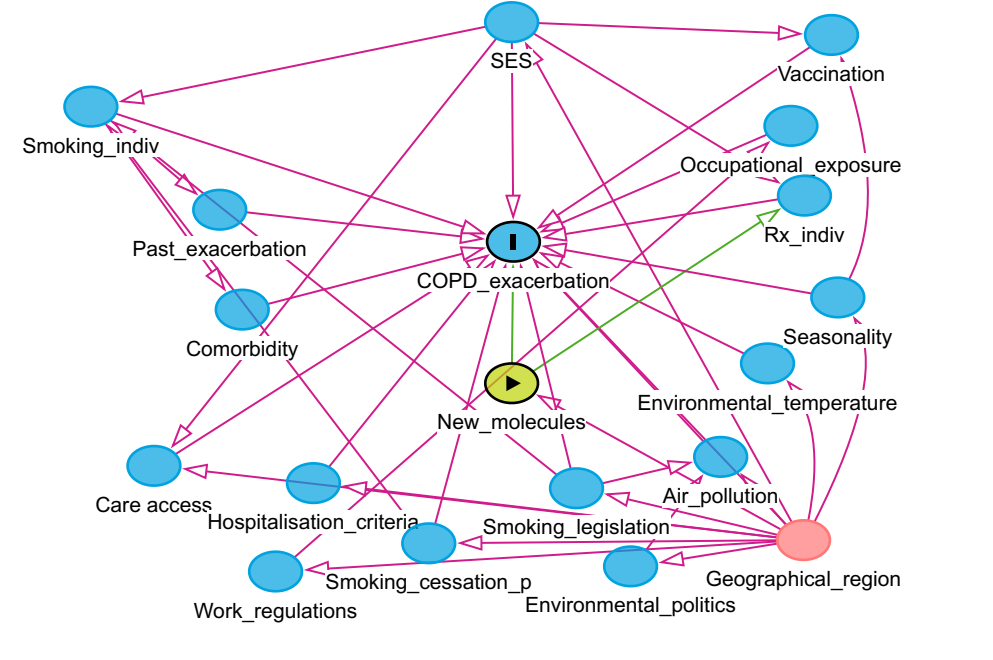 | ^30-34^ |
| Environmental Temperature | 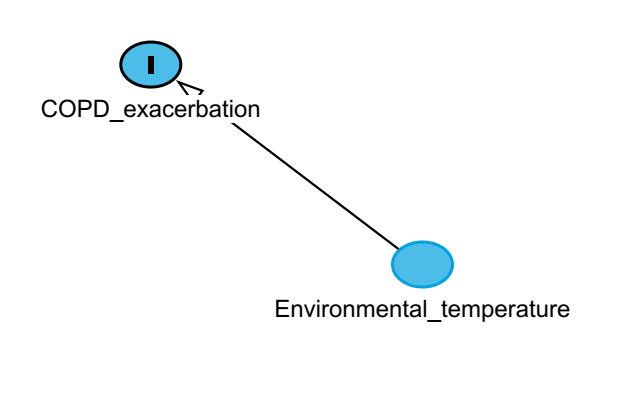 | ^31,32^ |
| Air pollution | 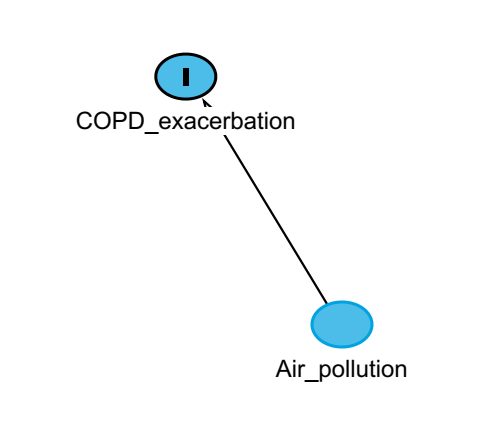 | ^29,30,34^ |

**Supplementary Figure 2: Severe AE-COPD risk factors and causal pathways.**

A directed acyclic graph (DAG) of the relationship between the introduction of ultra-LABAs/newer LAMAs and FDCs and severe COPD exacerbations has been constructed using DAGitty to try to identify sources of confounding bias^35^. Possible confounding effect of the population’s age, sex, past exacerbations, comorbidities prevalence, medications for COPD (including inhaled corticosteroids), socioeconomic status, social deprivation and region of residence monthly distributions were accounted for in the segmented regression models (**Supplementary Figure2**). Other individual level confounders identified graphically were not included in the statistical analyses because of their unavailability in the data sources (e.g., smoking status, COPD severity, occupational exposure and vaccination status). However, their impact on internal validity should be small due to their tendency to change only slowly over time^36,37^.


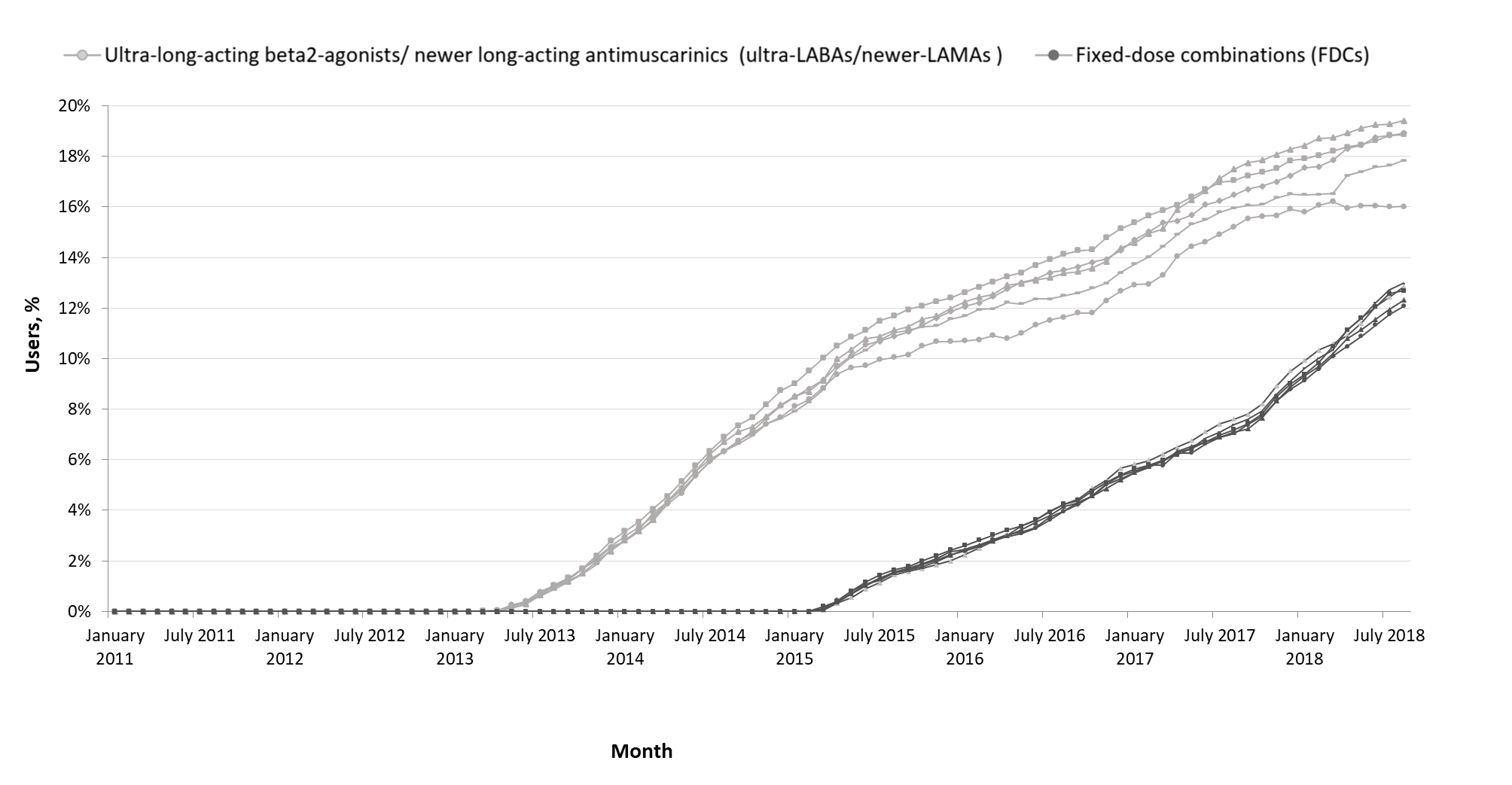


**Supplementary Figure 3: Proportion of ultra-LABAs/newer LAMAs and FDC users between April 2007 and August 2018 according to socioeconomic status groups.**

● 1^st^ quintile of material deprivation (least deprived); **-** 2^nd^ quintile of material deprivation; ▴3^rd^ quintile of material deprivation; ^◼^ 4^th^ quintile of material deprivation; ⬪ 5^th^ quintile of material deprivation (most deprived).


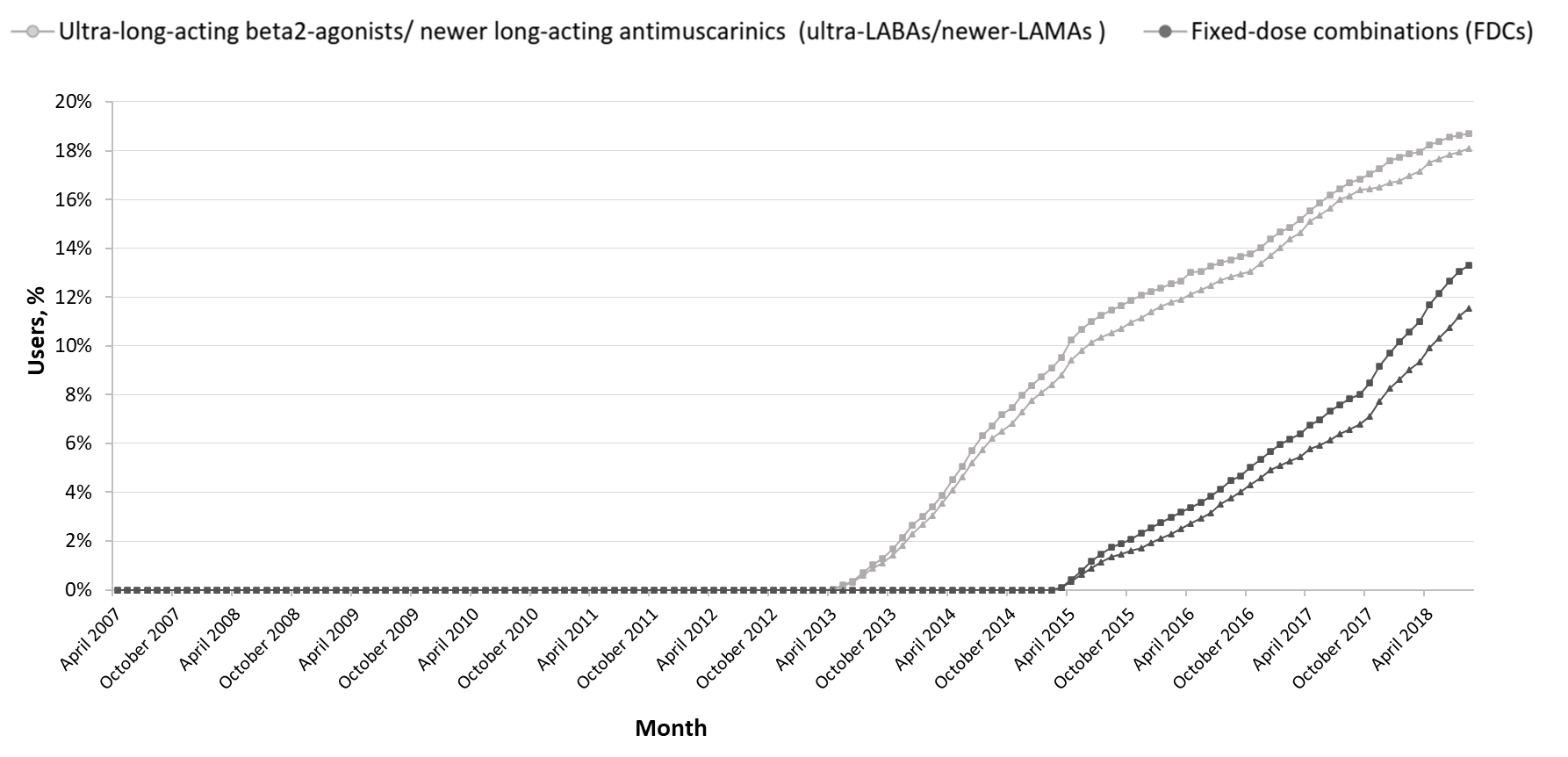


**Supplementary Figure 4: Proportion of ultra-LABAs/newer LAMAs and FDC users between April 2007 and August 2018 according to sex.**

▴Women; ^◼^ Men


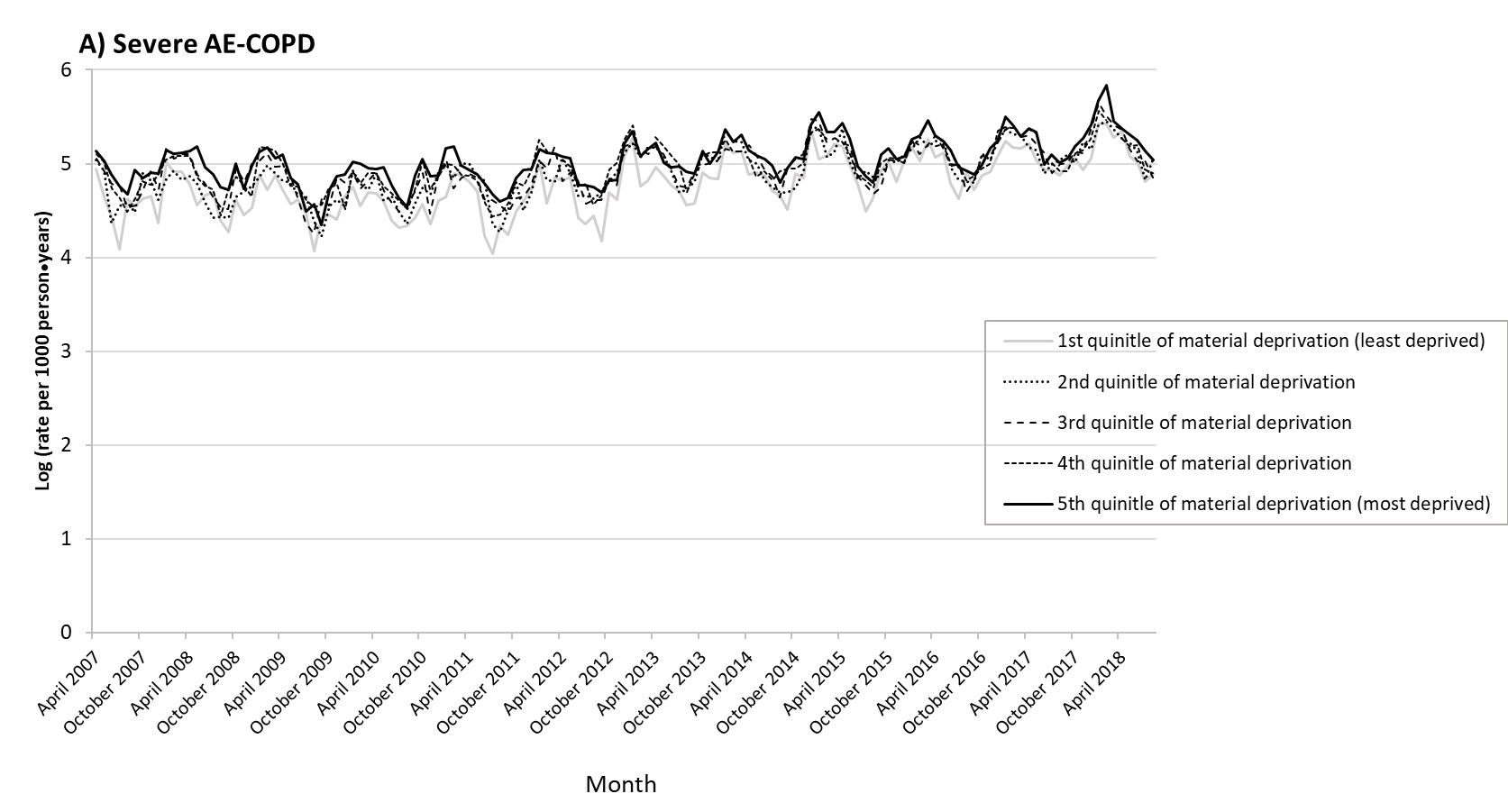


ultra-LABAs/newer-LAMAs’ introduction

FDCs’ introduction


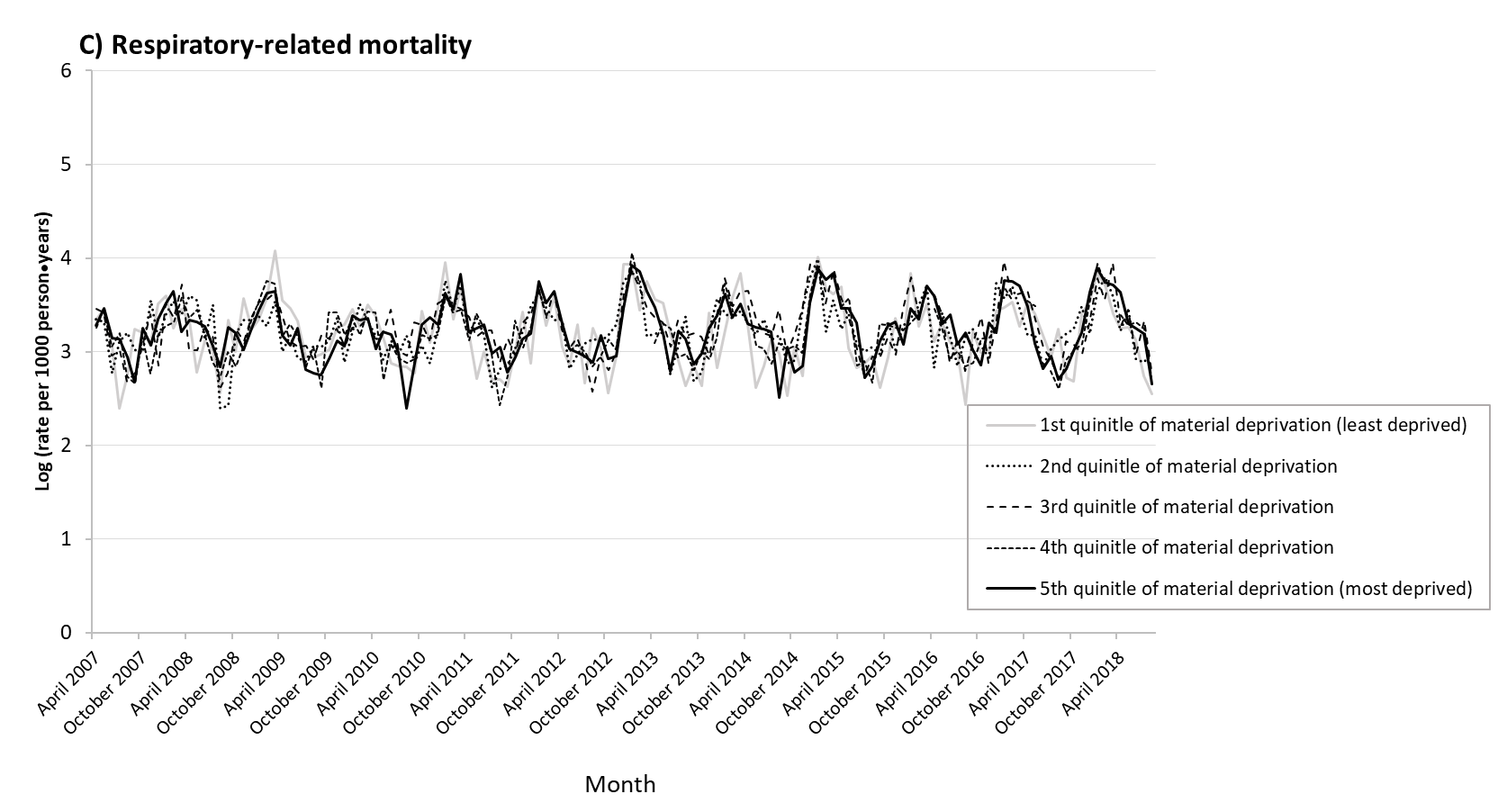

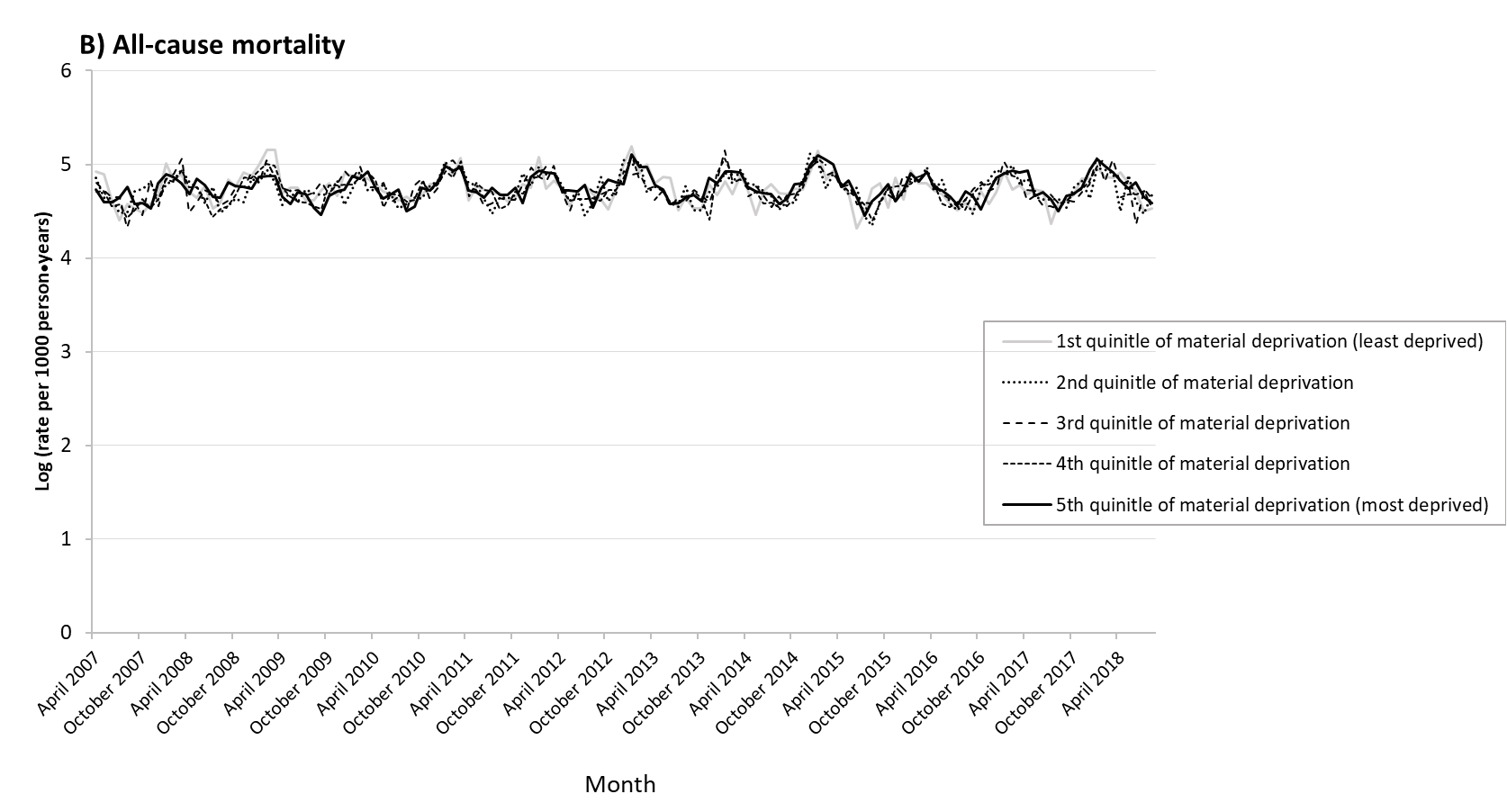


ultra-LABAs/newer-LAMAs’ introduction

FDCs’ introduction

ultra-LABAs/newer-LAMAs’ introduction

FDCs’ introduction

**Supplementary Figure 5: Severe AE-COPD and mortality trends by socioeconomic status.** Trends in A) severe AE-COPD, B) all-cause and, C) respiratory-related mortality gross rates are presented according to socioeconomic groups. Vertical dashed lines indicate March 2013 and February 2015 when ultra-LABAs/newer LAMAs and FDCS were introduced in Quebec, Canada, respectively.


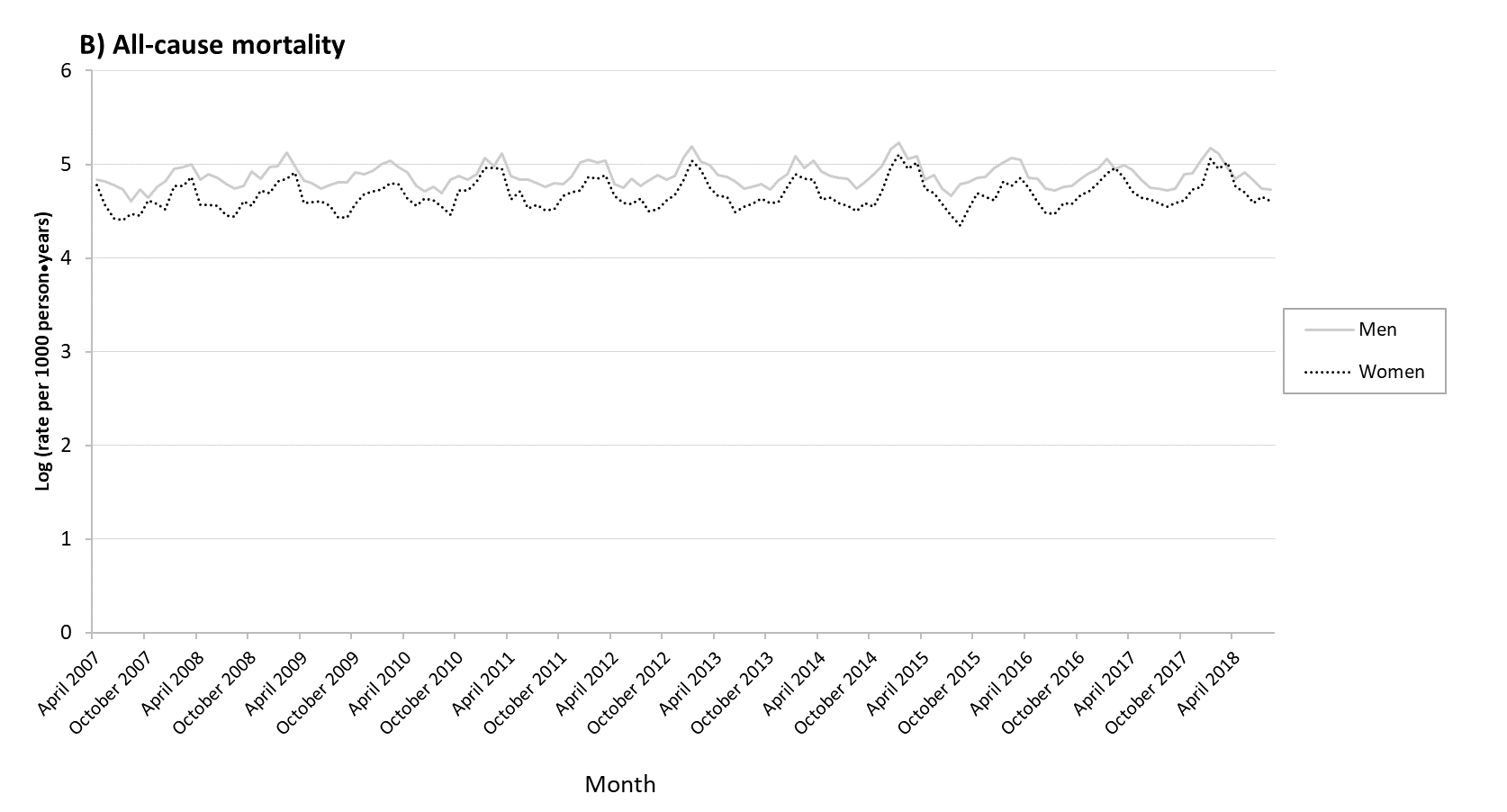

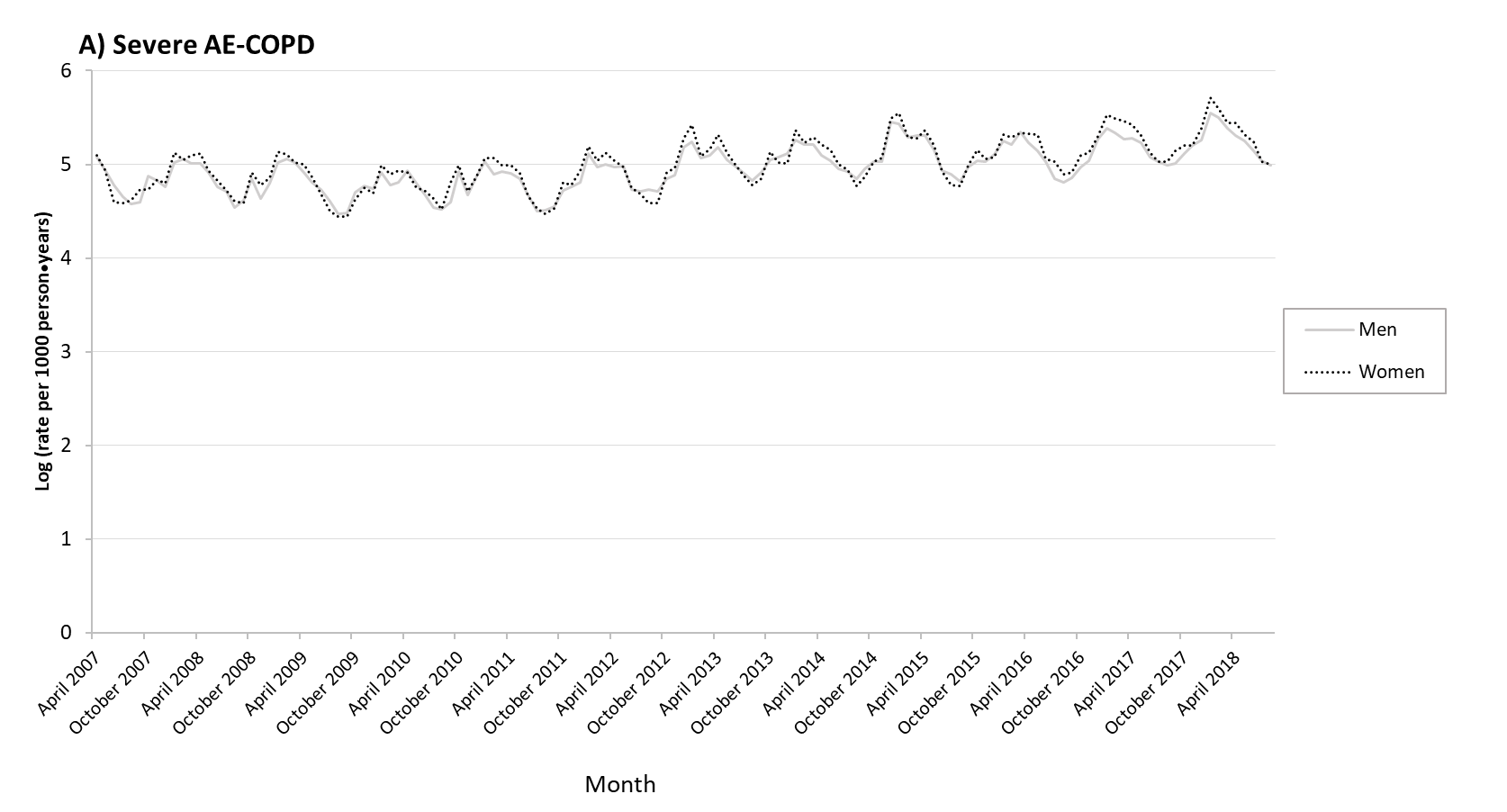


ultra-LABAs/newer-LAMAs’ introduction

FDCs’ introduction

ultra-LABAs/newer-LAMAs’ introduction

FDCs’ introduction


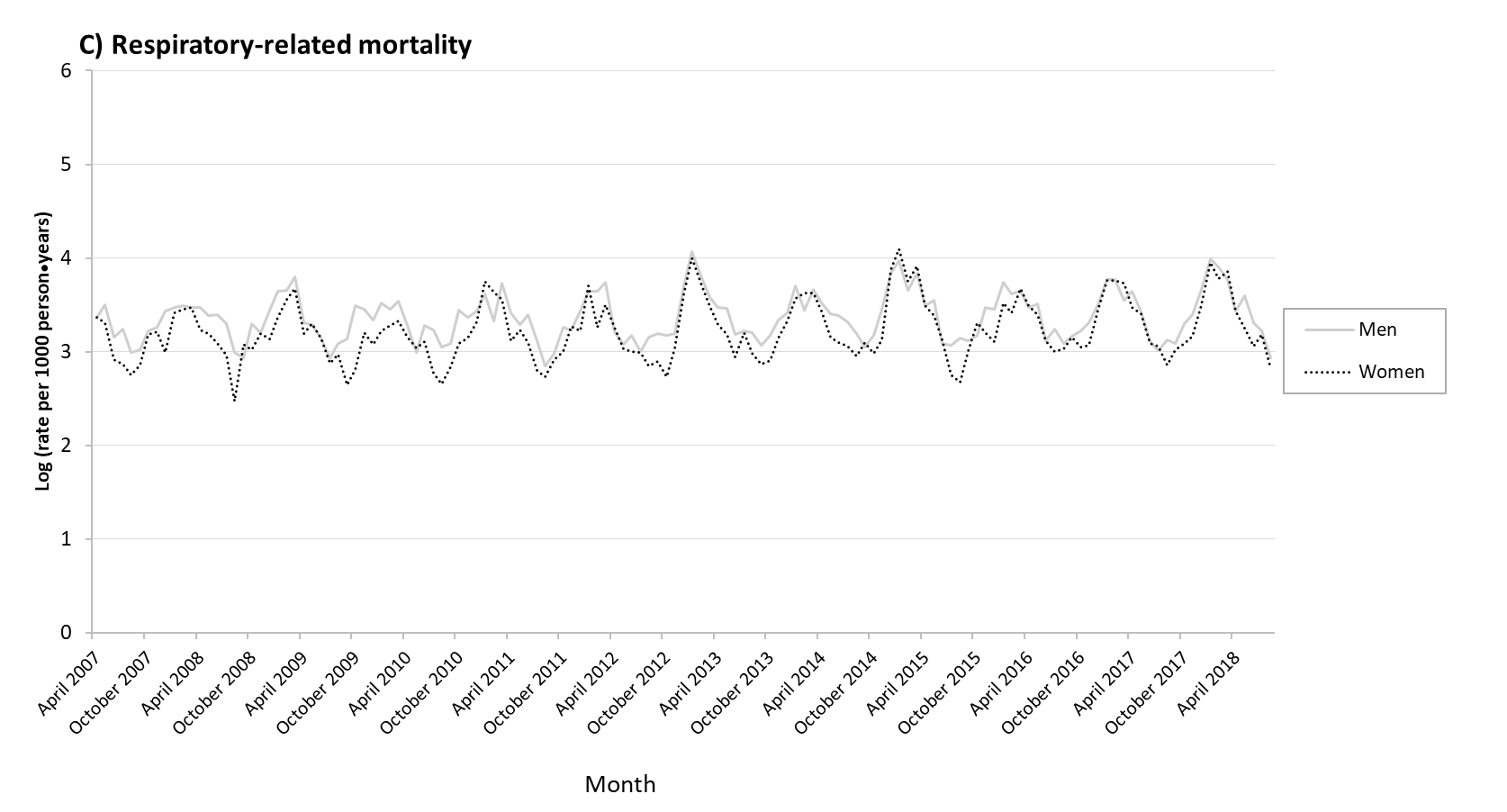


FDCs’ introduction

ultra-LABAs/newer-LAMAs’ introduction

**Supplementary Figure 6: Severe AE-COPD and mortality trends according to sex.** Trends in A) severe AE-COPD, B) all-cause and, C) respiratory-related mortality gross rates are presented according to sex. Vertical dashed lines indicate March 2013 and February 2015 when ultra-LABAs/newer LAMAs and FDCS were introduced in Quebec, Canada, respectively.

**Supplementary References**

E1. Lopez Bernal J, Cummins S, Gasparrini A. The use of controls in interrupted time series studies of public health interventions. Int J Epidemiol. 2018;47(6):2082-2093.

E2. Simard M, Sirois C, Candas B. Validation of the Combined Comorbidity Index of Charlson and Elixhauser to Predict 30-Day Mortality Across ICD-9 and ICD-10. *Med Care*. 2018;56(5):441-447.

E3. Hurst JR, Vestbo J, Anzueto A, et al. Susceptibility to exacerbation in chronic obstructive pulmonary disease. *N Engl J Med*. 2010;363(12):1128-1138.

E4. Beeh KM, Glaab T, Stowasser S, et al. Characterisation of exacerbation risk and exacerbator phenotypes in the POET-COPD trial. *Respir Res*. 2013;14:116.

E5. Niewoehner DE, Lokhnygina Y, Rice K, et al. Risk indexes for exacerbations and hospitalizations due to COPD. *Chest*. 2007;131(1):20-28. doi:10.1378/chest.06-1316

E6. Hartley BF, Barnes NC, Lettis S, Compton CH, Papi A, Jones P. Risk factors for exacerbations and pneumonia in patients with chronic obstructive pulmonary disease: a pooled analysis. *Respir Res*. 2020;21(1):5.

E7. Au DH, Bryson CL, Chien JW, et al. The effects of smoking cessation on the risk of chronic obstructive pulmonary disease exacerbations. *J Gen Intern Med*. 2009;24(4):457-463.

E8. Collins PF, Stratton RJ, Kurukulaaratchy RJ, Elia M. Influence of deprivation on health care use, health care costs, and mortality in COPD. *Int J Chron Obstruct Pulmon Dis*. 2018;13:1289-1296.

E9. Wong AWM, Gan WQ, Burns J, Sin DD, van Eeden SF. Acute exacerbation of chronic obstructive pulmonary disease: influence of social factors in determining length of hospital stay and readmission rates. *Can Respir J*. 2008;15(7):361-364.

E10. Cho KH, Nam CM, Lee EJ, et al. Effects of individual and neighborhood socioeconomic status on the risk of all-cause mortality in chronic obstructive pulmonary disease: A nationwide population-based cohort study, 2002-2013. *Respir Med*. 2016;114:9-17.

E11. Sahni S, Talwar A, Khanijo S, Talwar A. Socioeconomic status and its relationship to chronic respiratory disease. *Adv Respir Med*. 2017;85(2):97-108.

E12. Prescott E, Lange P, Vestbo J. Socioeconomic status, lung function and admission to hospital for COPD: results from the Copenhagen City Heart Study. *Eur Respir J*. 1999;13(5):1109-1114.

E13. Wong AWM, Gan WQ, Burns J, Sin DD, van Eeden SF. Acute exacerbation of chronic obstructive pulmonary disease: influence of social factors in determining length of hospital stay and readmission rates. *Can Respir J*. 2008;15(7):361-364.

E14. Hoogendoorn M, Feenstra TL, Hoogenveen RT, Al M, Mölken MR. Association between lung function and exacerbation frequency in patients with COPD. *Int J Chron Obstruct Pulmon Dis*. 2010;5:435-444.

E15. González C, Servera E, Marín J. Importance of noninvasively measured respiratory muscle overload among the causes of hospital readmission of COPD patients. *Chest*. 2008;133(4):941-947.

E16. Tashkin DP, Celli B, Senn S, et al. A 4-year trial of tiotropium in chronic obstructive pulmonary disease. *N Engl J Med*. 2008;359(15):1543-1554.

E17. Van den Bruel A, Gailly J, Neyt M. Does tiotropium lower exacerbation and hospitalization frequency in COPD patients: results of a meta-analysis. *BMC Pulm Med*. 2010;10:50.

E18. Stockley RA, Whitehead PJ, Williams MK. Improved outcomes in patients with chronic obstructive pulmonary disease treated with salmeterol compared with placebo/usual therapy: results of a meta-analysis. *Respir Res*. 2006;7:147.

E19. Rennard SI, Calverley PMA, Goehring UM, Bredenbröker D, Martinez FJ. Reduction of exacerbations by the PDE4 inhibitor roflumilast--the importance of defining different subsets of patients with COPD. *Respir Res*. 2011;12:18.

E20. Calverley PMA, Rabe KF, Goehring U-M, et al. Roflumilast in symptomatic chronic obstructive pulmonary disease: two randomised clinical trials. *Lancet*. 2009;374(9691):685-694.

E21. Vogelmeier C, Hederer B, Glaab T, et al. Tiotropium versus salmeterol for the prevention of exacerbations of COPD. *N Engl J Med*. 2011;364(12):1093-1103.

E22. Almagro P, Cabrera FJ, Diez J, et al. Comorbidities and short-term prognosis in patients hospitalized for acute exacerbation of COPD: the EPOC en Servicios de medicina interna (ESMI) study. *Chest*. 2012;142(5):1126-1133.

E23. Xu W, Collet J-P, Shapiro S, et al. Independent effect of depression and anxiety on chronic obstructive pulmonary disease exacerbations and hospitalizations. *Am J Respir Crit Care Med*. 2008;178(9):913-920.

E24. Jencks SF, Williams MV, Coleman EA. Rehospitalizations among patients in the Medicare fee-for-service program. *N Engl J Med*. 2009;360(14):1418-1428.

E25. Lin J, Xu Y, Wu X, et al. Risk factors associated with chronic obstructive pulmonary disease early readmission. *Curr Med Res Opin*. 2014;30(2):315-320.

E26. Furumoto A, Ohkusa Y, Chen M, et al. Additive effect of pneumococcal vaccine and influenza vaccine on acute exacerbation in patients with chronic lung disease. *Vaccine*. 2008;26(33):4284-4289.

E27. Kiyohara K, Kojimahara N, Sato Y, Yamaguchi N. Changes in COPD mortality rate after amendments to the Preventive Vaccination Law in Japan. *Eur J Public Health*. 2013;23(1):133-139.

E28. Poole PJ, Chacko E, Wood-Baker RW, Cates CJ. Influenza vaccine for patients with chronic obstructive pulmonary disease. *Cochrane Database Syst Rev*. 2000;(4):CD002733.

E29. de Miguel-Díez J, Hernández-Vázquez J, López-de-Andrés A, Álvaro-Meca A, Hernández-Barrera V, Jiménez-García R. Analysis of environmental risk factors for chronic obstructive pulmonary disease exacerbation: A case-crossover study (2004-2013). *PLoS One*. 2019;14(5):e0217143.

E30. DeVries R, Kriebel D, Sama S. Low level air pollution and exacerbation of existing copd: a case crossover analysis. *Environ Health*. 2016;15(1):98.

E31. Jenkins CR, Celli B, Anderson JA, et al. Seasonality and determinants of moderate and severe COPD exacerbations in the TORCH study. *Eur Respir J*. 2012;39(1):38-45.

E32. Donaldson GC, Seemungal T, Jeffries DJ, Wedzicha JA. Effect of temperature on lung function and symptoms in chronic obstructive pulmonary disease. *Eur Respir J*. 1999;13(4):844-849.

E33. Holt JB, Zhang X, Presley-Cantrell L, Croft JB. Geographic disparities in chronic obstructive pulmonary disease (COPD) hospitalization among Medicare beneficiaries in the United States. *Int J Chron Obstruct Pulmon Dis*. 2011;6:321-328.

E34. Croft JB, Wheaton AG, Liu Y, et al. Urban-Rural County and State Differences in Chronic Obstructive Pulmonary Disease - United States, 2015. *MMWR Morb Mortal Wkly Rep*. 2018;67(7):205-211.

E35. Textor J, van der Zander B, Gilthorpe MS, Liśkiewicz M, Ellison GT. Robust causal inference using directed acyclic graphs: the R package ‘dagitty.’ *International Journal of Epidemiology*. 2016;45(6):1887-1894.

E36. Bondy SJ, Victor JC, Diemert LM, et al. Transitions in Smoking Status Over Time in a Population-Based Panel Study of Smokers. *Nicotine & Tobacco Research*. 2013;15(7):1201-1210.

E37. De Oliveira Bernardo C, González-Chica DA, Chilver M, Stocks N. Influenza immunisation coverage from 2015 to 2017: A national study of adult patients from Australian general practice. *Vaccine*. 2019;37(31):4268-4274.
